# Improved Statistical Analysis of Low Abundance Phenomena in Bimodal Bacterial Populations

**DOI:** 10.1371/journal.pone.0078288

**Published:** 2013-10-30

**Authors:** Friedrich Reinhard, Jan Roelof van der Meer

**Affiliations:** Department of Fundamental Microbiology, University of Lausanne, Lausanne, Switzerland; University of Illinois, United States of America

## Abstract

Accurate detection of subpopulation size determinations in bimodal populations remains problematic yet it represents a powerful way by which cellular heterogeneity under different environmental conditions can be compared. So far, most studies have relied on qualitative descriptions of population distribution patterns, on population-independent descriptors, or on arbitrary placement of thresholds distinguishing biological ON from OFF states. We found that all these methods fall short of accurately describing small population sizes in bimodal populations. Here we propose a simple, statistics-based method for the analysis of small subpopulation sizes for use in the free software environment *R* and test this method on real as well as simulated data. Four so-called population splitting methods were designed with different algorithms that can estimate subpopulation sizes from bimodal populations. All four methods proved more precise than previously used methods when analyzing subpopulation sizes of transfer competent cells arising in populations of the bacterium *Pseudomonas knackmussii* B13. The methods’ resolving powers were further explored by bootstrapping and simulations. Two of the methods were not severely limited by the proportions of subpopulations they could estimate correctly, but the two others only allowed accurate subpopulation quantification when this amounted to less than 25% of the total population. In contrast, only one method was still sufficiently accurate with subpopulations smaller than 1% of the total population. This study proposes a number of rational approximations to quantifying small subpopulations and offers an easy-to-use protocol for their implementation in the open source statistical software environment *R*.

## Introduction

Advances in microbiology have traditionally been based on studies at the population level. Questions of how cells respond to their environment, interact with each other, or undergo complex processes such as cellular differentiation or gene expression have been mostly answered by inference from population-level data. Recent technological advances have facilitated the study of individual cells and led to new appreciation for the existence and importance of phenotypic heterogeneity [1], [2]. There is no more doubt that gene expression is heterogeneous among cells in isogenic microbial populations and leads to physiological heterogeneity [3]–[5]. In many cases distributions of physiological parameters among individual cells in populations show a small part, usually less than a few percent of the total, to be more than two-fold different from the population average [6]–[8]. It is thought that the appearance or existence of small subpopulations with different phenotypes in a clonal population may be beneficial for its survival under adverse conditions [5], [9]. As example, persistence to antibiotic toxicity in *Escherichia coli* is a very rare phenomenon [10], yet it is of great importance since it enables population survival and outgrowth when the antibiotic is removed. Growth to stationary phase of *B. subtilis* leads to the appearance of subpopulations with widely varying expression of glycolysis and gluconeogenesis enzymes that are thought to better enable stationary phase survival [5]. In fact, an increasing number of phenotypic traits has been discovered that are not even homogenously distributed among all cells in a clonal bacterial population but rather lead to the formation of two (bimodal) distinct subpopulations. Current examples from microbiology include horizontal gene transfer activation in *Pseudomonas* [11]–[13], sporulation [14], [15], cannibalism [16], extracellular matrix formation [17], competence development [18], [19], and motility [20], [21] in *Bacillus subtilis*, the lysis-lysogeny switch of phage lambda [22], lactose utilization [23], the arabinose catabolic pathway [24], and chemotaxis in *E. coli* [25], quorum sensing-regulated bioluminescence in *Vibrio harveyi* [26], flagella expression in *Salmonella* Typhi [27], or phase variation in a number of pathogens [28], [29]. There is no reason not to assume that many more and diverse bimodal or even multimodal phenotypic differentiations in clonal bacterial populations would exist, and there is evidence that the extent of phenotypic variability is a selectable trait [4]. Evidently, in order to better understand bimodal phenomena it is of critical importance to have accurate measurement and analysis tools for differentiating subpopulations within the total population. Most authors exploring bimodal phenomena have been relying on production of autofluorescent proteins to study critical promoters and regulatory events at the single cell level, mainly because of the ease to detect expression of the reporter protein in individual cells [3], [4], [30]. Such detection is typically performed by either epifluorescence microscopy and digital image analysis [3], [5], [9], [31]–[33] or by flow cytometry, if expression of the fluorescent reporter protein is sufficiently high [4], [17], [34]–[38]. Measures of expression heterogeneity such as occurrence of bimodalities or subpopulation sizes, represent useful parameters to quantify phenotypic heterogeneity and its differences in mutants or as a result of growth conditions. However, the more one approaches very small subpopulation sizes (e.g., a few percent of the total) the more difficult it is to accurately detect and determine such events, and so far most methods do not take such low proportions into appropriate consideration. For example, subpopulation dynamics is often solely assessed in form of descriptive graphs that present the total distribution of fluorescence intensities for individuals. These included histograms [5], [23], [24], [34], [35], [39], cumulative distribution curves (CDFs) [19], [40], [41] [42], [43], normal quantile-quantile (Q-Q) plots [30], [44], [45] or percentile-percentile (P-P) plots [46]. Although representations of total populations are useful for stating evident differences in distribution patterns between treatments, they tend to overlook more subtle differences which often need a quantitative approach. Quantification of subpopulation dynamics is generally done by addressing individual fluorescence values that fall within pre-defined boundaries of the total population. However, often these boundaries are determined independently of the nature of the distribution of the total population data. An example of this is when gating of clusters in flow cytometry is manually defined to identify subpopulation shifts [17], [36], [38], [47] or when threshold rules are based on background or control fluorescence in fluorescence microscopy to determine “all-or-none” induction responses [11], [21], [26], [33], [37]. A problem with subpopulation quantification using pre-defined and distribution-independent thresholding is that such classification does not attempt to statistically approximate estimates for true, that is biologically relevant, subpopulations (since boundaries have nothing or little to do with the distribution of the data), but rather represent a pragmatic approach to achieve differentiation between treatments. Therefore, generally, such approach falls short of serving as a universal method for subpopulation quantification, especially when subpopulations overlap. One solution to this problem would entail a distribution-based approximation of the distinct subpopulations that is entirely independent of the experimental test system used (as long as the test system is sensitive enough), and the result of which could be expressed as a dimensionless quantity.

The aim of this study is to propose a methodology for quantifying small subpopulations (few percent) in bimodal populations. Our approach is based on a statistically valid approximation to accurately estimate the “true” subpopulation size in bimodal populations and expressing it as a percentage of the total population size. The model system we use to develop our method is the bistable behaviour of the integrative and conjugative element called ICE*clc* of the bacterium *Pseudomonas knackmussii* B13 [40], [48]–[50]. It was previously discovered that the promoter of the integrase gene (P_int_) on ICE*clc* expresses under stationary-phase conditions in some 3% of cells in culture, specifically when they have been grown with 3-chlorobenzoate (3CBA) as sole carbon and energy source [40], [51]. Cells that induce P_int_ are locked in a bistable state [11] and undergo a process of competence formation which enables ICE*clc* transfer [13]. ICE*clc* behaviour was inferred from single-cell fluorescence measurements on strains carrying an additional single-copy transcriptional fusion between P_int_ and the gene for enhanced green fluorescent protein (eGFP) or mCherry. In first instance and because of the absence of clear bimodality, distribution-independent descriptors were used to describe P_int_ expression [40], [51]. For that purpose, eGFP fluorescence intensities of at least one thousand imaged cells were ranked, from which the 95th percentile and the mean fluorescence intensity among the top five percent were calculated [40], [51]. Alternatively, subpopulation sizes were determined from the ‘breakpoint’ in cumulatively ranked fluorescence values of thousands of individual imaged cells [12]. Here we evaluate different methods for subpopulation characterization and propose a simple routine in the open source statistical software *R* that integrates some of the ideas of earlier studies [11], [12], [30]. As these methods require population splitting (PS) into a large and small subpopulation (by use of a cutoff value) we call them PS methods. Our PS methods are particularly suitable for analysis of subpopulations of only a few percent of the total, which may otherwise be difficult to discern. A first data verification step is incorporated in the subroutine that summarizes data from different images to ensure that no outlier exposure errors or biases exist. The following steps then help to find the statistically most likely appropriate subpopulation size. We challenge PS methods in two ways; firstly, by measuring subpopulation sizes of ICE*clc* transfer competent cells of *P. knackmussii* B13 under different growth conditions, and secondly, by quantifying subpopulation sizes of computer-generated mixed populations.

## Methods

### Culture Conditions

All strains in this work are listed in Table S1. All strains were batch-cultured in 200 ml Erlenmeyer flasks containing 30 ml liquid minimal at 30°C and with 200 rpm rotary shaking. Type 21C minimal medium (MM) [52] was supplemented with either 3CBA (10 mM), fructose (10 mM), glucose (10 mM), benzoate (10 mM), anthranilate (10 mM) or 4-hydroxybenzoate (10 mM) as sole carbon and energy source. Increase in culture turbidity at 600 nm was followed during growth to estimate the onset of the stationary phase and to define exact sampling times for epifluorescence microscopy (Table S2). Stationary phase samples (i.e., 10 to 30 hours after cessation of turbidity increase in batch culture) of three microliter were deposited on microscope glass slides, covered with a 0.17 mm cover slip and immediately imaged.

### Promoter Reporter Gene Fusions

To examine expression of the P_int_ promoter at single cell level we used previously constructed transcriptional fusions between P_int_ and promoterless *egfp* genes [11], that were inserted in single copy on the chromosome of a variety of *Pseudomonas* strains (Table S1) via mini-Tn5 delivery, and verified by antibiotic selection markers and specific PCR amplification.

### Digital Imaging

Fluorescence intensities of single cells with or without transcriptional fusions to the *egfp* gene were determined by digital imaging. Single cells were visualized at 1000-fold magnification under a Zeiss Axioscope2 upright epifluorescence microscope equipped with a Spot Xplorer 1.4 MPixel cooled CCD camera (Visitron Systems GmbH, Puchheim, Germany). Images were recorded with phase-contrast illumination (10 ms) and with the filter eGFP HQ470/40 for eGFP fluorescence (excitation wavelength 480±20 nm, emission wavelength 520±20 nm, 500 ms) (Chroma Technology Corp, VT, USA). Average intensity values (AGV) of each cell were determined from 16-bit stored TIF-images using the program Metaview (version 6.1r5, Visitron Systems GmbH) using the phase-contrast image as mask for outlining the cells in the eGFP channel. Data were exported to Excel (Microsoft Corporation, Redmond, Wash.) or *R* [53]. At least 1000 cells were measured for each condition and at least six images were taken per condition or strain.

### Programming in *R*

All statistical analysis and computations were processed in *R*. For PS methods, an approach was followed that assumed bimodality of the data (i.e., containing two subpopulations each with a normal distribution). The list of individual cellular AGVs was hereto transferred from Excel to a data text file, which was placed into an *R* work-folder. Data were processed according to different PS and non-PS methods in a subroutine written in *R* named *findsub(…)* (Protocol S1, S2). Essentially, the setting *Default* in *findsub(…)* ranks data according to their AGV and plots the values against a theoretical normal distribution (the normal Q-Q plot) (Protocol S1) [30]. Subsequently, the subroutine determines the median and a region around the median to produce the linear regression line for the larger subpopulation. A horizontal separator line is then automatically generated according to 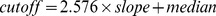, where *cutoff* is the point at which the horizontal separator line is drawn, *slope* is slope of the linear regression line (and therefore the standard deviation of the large subpopulation), and *median* is the median of the data set (Protocol S1). All data points above the horizontal separator line are considered to belong to the smaller subpopulation. The subroutine in *Default* mode further allows manual setting of the range of the large population from which the median value is determined via mouse-clicking on an interactive graph (Protocol S1). Other PS modes of *findsub(…)* include the modes *Manual*, *Boxplot1.5* and *Boxplot3*. While *Manual* allows manual determination of the breakpoint between subpopulations via mouse-clicking on an interactive graph (Protocol S1, Figure S3), *Boxplot1.5* and *Boxplot3* use an outlier algorithm as calculated by the *R* function *boxplot(…)* (*R* graphics package) (Protocol S1). The argument *range* of the function *boxplot(…)* determines how far the plot whiskers extend out from the box beyond which outliers are identified. *Boxplot1.5* uses *range = *1.5 and *Boxplot3* uses *range* = 3, corresponding to mild and extreme outlier detection, respectively [54]. Finally, a fifth mode of *findsub(…)* is the mode *Other*. This mode calculates results according to four non-PS algorithms including the population mean (*Mean*), and the population-independent methods 95^th^ percentile (*95^th^ Percentile*), mean between the 75^th^ and 95^th^ percentile (*Boosted Mean*), and mean of the top 5% of a population (*Mean Top 5%*) (Protocol S1).

Finally, the subroutines *get.ci(…)* and *get.ci.other(…)* were written in *R* (Protocol S2), allowing to bootstrap PS and non-PS methods, respectively, for 95% confidence interval determination. Bootstrapping was carried out via random sampling with replacement of data sets with subsequent application of the method of choice with n repetitions (Protocol S1, S2). For confidence interval calculations with 20 repetitions (*Default, Manual*), a normal distribution of the bootstrapped results was assumed (Protocol S1): 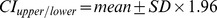, where *CI_upper/lower_* is the upper or lower confidence interval, respectively, *mean* is the population mean and *SD* is standard deviation. For methods *Default* and *Manual* repetitions were limited to 20 because every calculation requires manual intervention on an interactive graph for the method to work.

For confidence interval calculations with 500 repetitions (all other methods), the *R* function *boot.ci(…)* from the *R* boot package [55], [56] was used with the percentile method of bootstrap confidence interval calculation.

### Simulations and Data Presentation

Bimodal populations were simulated by mixing a large subpopulation with multiple, smaller subpopulations varying in standard deviation, mean and size, respectively. Large and small subpopulations were created with the function *rnorm(…)* of the *R* statistical package [53]. Parameters for the creation of the large subpopulation were set to standard deviation *SD* = 3.9, and mean *mean* = 63, both of which were considered typical values for AGV data sets obtained from stationary phase batch cultures of ICE*clc*-harbouring *Pseudomonas* tagged with a P_int_ *-egfp* reporter and grown on 3CBA (Table S3). Size *N* of the mixed populations was set to 2000, 20000 or 200000. Parameters for the creation of subpopulations were set to all possible combinations of either 40 or 15 equidistantly spaced values for standard deviations, mean values, or population sizes, which in total yielded 40^3^ ( = 64000) or 15^3^ ( = 3375) different subpopulations, respectively. The ranges for 40 equidistantly spaced parameter values were set to 10 to 50 for standard deviations, 65 to 200 for mean values, and 0.1% to 40% of the total population for small subpopulation sizes. The ranges for 15 equidistantly spaced parameter values were set as above except for 0.1% to 1.2% for small subpopulation sizes. Small subpopulation determination was carried out according to the PS methods *Boxplot1.5* and *Boxplot3*. For code and script for the simulation of mixed populations and their separation using *Boxplot1.5* and *Boxplot 3* see Protocol S3. The *R* package “lattice” [57] was used for 3D visualizations of the data by use of the function *wireframe(…)*. The freeware ImageJ (version 1.440, USA) was used for creating movies of the visualisations (Video S1–S9).

## Results

### Stationary Phase Induction of P_int_-*egfp* in *P. knackmussii* B13

**Figure 1:**
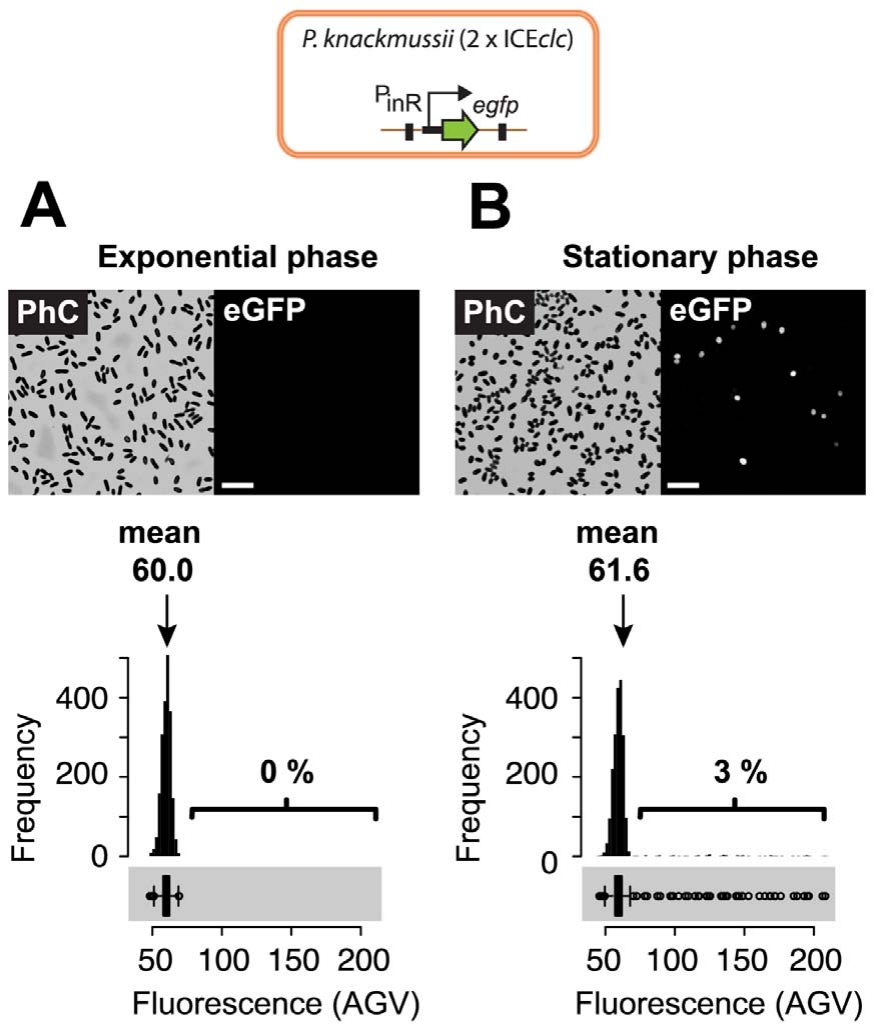
eGFP expression from a monocopy randomly inserted P_inR_-*egfp* fusion in planctonic cells of *P. knackmussii* B13 grown in batch culture and sampled in exponential phase (A) or stationary phase (B). Micrographs show typical population differences of cells grown on 5-chlorobenzoate (3CBA) under non-inducing (exponential phase) and inducing conditions (stationary phase), taken under eGFP illumination (right) and the corresponding image in phase contrast (PhC, left). The white bar in images corresponds to a scale of 10 µm. Graphs show fluorescence values (AGVs) measured from single cells represented as histograms and lateral boxplots (grey area below graph). Percentages correspond to calculated sizes of subpopulations statistically significantly expressing eGFP. Note that the calculated mean fluorescence values over the whole population are statistically significantly different if assuming both are normally distributed (P = 0.00056, Welch two-sample t-test).

**Figure 2:**
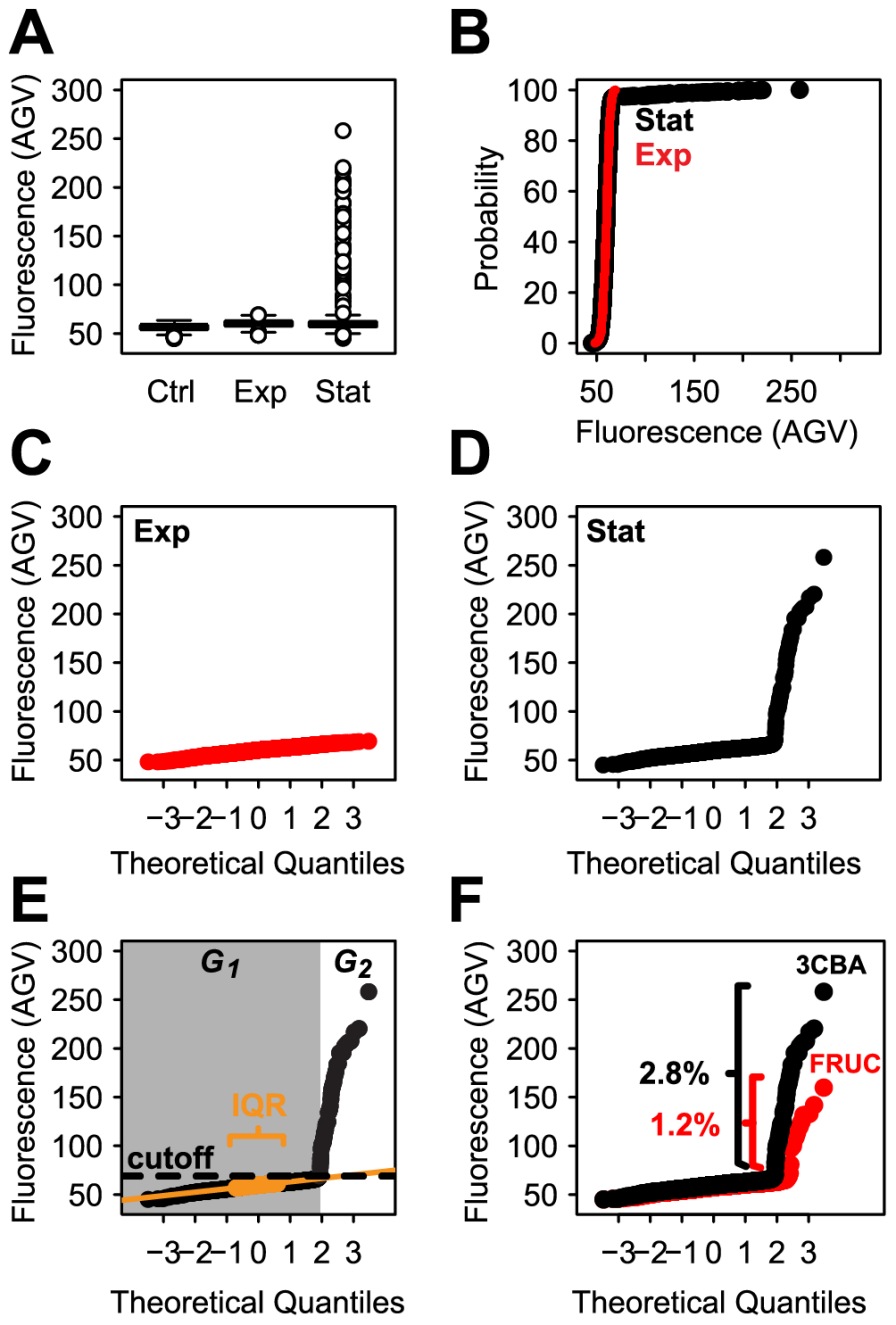
Distribution of eGFP fluorescence intensities (AGV) in cells of *P. knackmussii* B13 strain 1343 (single copy insertion of a P_int_-*egfp* fusion) taken at exponential or stationary phase after growth on 10 mM 3CBA. (A) Boxplot representation. Ctrl, wildtype *P. knackmussii* B13 without eGFP. (B) Cumulative distribution curve representation (CDF). Stat, stationary phase. Exp, exponential phase. (C, D) Same as (B) but as normal quantile-quantile (Q-Q) plot representations. (E) Same as (D) but data is categorized in large subpopulation (*G_1_*, grey area) and small subpopulation (*G_2_*, white area) below and above a cutoff line (cutoff), respectively. The placement of the cutoff line is determined via the slope of a regression line (orange line) fitted to the data points belonging to the interquartile range (IQR, orange) of the large subpopulation. (F) Distribution of eGFP fluorescence intensities in cells taken at inducing conditions (stationary phase) grown on either 10 mM 3CBA (CBA, black) or 10 mM fructose (Fruc, red). Percentages express subpopulation fractions of fructose and 3CBA induced cells (see further Table 1).

Single cell fluorescence can be quantified from a digital image with the help of image analysis software that recognizes cells as objects through thresholding of pixel intensities, and measures their average pixel fluorescence intensity (AGV). AGVs of all cells are typically plotted as histograms, CDFs, or as Q-Q plots. As noticed previously [11], [40], cells of *P. knackmussii* B13 P_int_-*egfp* did not visibly fluoresce during exponential growth on 3CBA, whereas a small proportion of cells in the culture induced *egfp* in stationary phase (Figure 1A and 1B). This difference is reflected in the shapes of the histograms that can be constructed from the AGVs of cells grown under these conditions; in the histograms of Figure 1 both populations look similarly in that they follow the shape of a normal distribution. However, paying attention to detail, it can be seen that under stationary phase conditions, a small proportion of cells manifests as a far-stretched right-hand tail of the histogram (Figure 1B and lower boxplot), which under exponential phase conditions is missing (Figure 1A and lower boxplot). The eGFP expression of such cells could be considered as outliers, or they could comprise a separate subpopulation, in which case the distribution of the data would be bimodal. The distribution is visualized more clearly in a boxplot representation, where, under stationary phase conditions, the histogram upper tail corresponds to boxplot outliers (Figure 1B, 2A). A CDF shows this particular subpopulation of cells with high eGFP expression as a ‘kink’ (Figure 2B, also see [40]), while in a normal Q-Q plot two lines with different slopes can be seen (Figure 2D, also see [30]). In all representations it becomes apparent that there is a subpopulation of cells behaving differently, but the Q-Q plot representation indicates that the data are bimodal. On the other hand, mean values alone, as commonly used as a measure in averaged samples, would not have revealed the bimodal nature of the population.

Which parameters would best describe and quantify the subpopulation effect? Quantification of the extent of bimodality is particularly important when less evident differences in population responses occur or effects of e.g., mutations need to be interpreted. For example, previous analysis suggested that P_int_-*egfp* is induced more strongly under stationary phase conditions when cells are pre-grown on 3CBA than on fructose [40]. This interpretation was based on use of distribution-free analyses and parameters such as the 95th percentile, the boosted mean or the mean of the top 5% of the population in a CDF [11], [40]. Although these methods have worked satisfactorily to conclude that cells that had grown on 3CBA were different from those grown on fructose [40], they did not provide a biological explanation for the choice of the 95% percentile-AGV value. Other distribution-free parameters like the boosted mean (mean of AGV between 75 and 95^th^ percentile) or mean of the top 5% AGV of the population also permitted statistical differentiation of eGFP expression from P_int_-*egfp* in cultures of *P. knackmussi* B13 under different growth conditions, but did not allow calculation of the actual subpopulation size [40]. Therefore, we decided to follow another approach that aimed to separate the bimodal data, which would allow the level of induction to be described in terms of the percentage of induced cells of the total population and mean AGV of induced cells. Because these methods rely on splitting of the population into large and small subpopulation, we refer to these methods as population splitting (PS) methods.

### Quantile-quantile Plot Interpretation of Bimodality

When plotting all AGV values in cumulative order as a function of their theoretically derived normally distributed ranking number, a so-called normal Q-Q plot, normally distributed AGV values among a population will become visible as a straight line (Figure 2C, also see [30], [46]), the slope of which corresponds to the standard deviation of the population. The median AGV in a normal Q-Q plot is found at the ranking number of ‘zero’ (Figure 2C–F). Deviations from a normal distribution will become visible in the normal Q-Q plot as deviations from the straight line (Figure 2D). Ideally, bimodal normally distributed subpopulations appear as two intersecting straight lines with different slopes (and therefore different standard deviations). Indeed, while AGV values of single cells in exponentially growing populations of *P. knackmussii* B13 cells expressing *egfp* from P_int_ were distributed along a single straight line (Figure 2C), AGVs from cells in stationary phase distributed in the diagram along two straight lines with different slopes (Figure 2D). Calculation of the size of the (eGFP inducing) smaller subpopulation would thus in essence consist of finding a statistically correct approximation of the point where the two straight lines would intersect and subsequent determination of the number of data points in each population. However, this proves difficult because it is impossible to determine *a priori* whether cells close to the intersection point would belong to one or the other subpopulation. Nevertheless, because of the large size of the ‘eGFP uninduced’ subpopulation (large subpopulation) compared to that of the eGFP inducing one (small subpopulation), a highly robust linear regression can be calculated for the large subpopulation on basis of a sub-sample of this subpopulation. We took this sub-sample as equivalent to the approximate interquartile range (IQR) (Figure 2E) of the large subpopulation. The large subpopulation IQR can be calculated from all AGV points between visually placed minimum and maximum AGVs (grey area: Figure 2E, Protocol S1), which can easily be estimated from a normal Q-Q plot. Since the slope in a Q-Q plot corresponds to the standard deviation it can be used to calculate the upper cutoff value at the 1% confidence level assuming that the large subpopulation is normally distributed (Figure 1E): 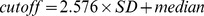, where 2.576 is the constant of the quantile function of the normal distribution with probability 0.995, *SD* is the standard deviation of the large subpopulation and *median* is the median of the large subpopulation. When applying such method, we calculated that 2.8% of cells in stationary phase cultures of *P. knackmussii* B13 P_int_-*egfp* grown on 3CBA and 1.2% in cultures grown on fructose expressed *egfp* statistically different from the large subpopulation (Figure 2F, Table 1). The method, therefore, permitted calculation of subpopulations of proportionally low abundance (≈ few percent of the total).

**Table 1:** Varying subpopulation sizes of ICE*clc* transfer competent cells in *P. knackmussii* B13–1343 P_int_ *-egfp* grown on different carbon sources.

Category	Carbon source^1^	% Subpopulation^2^	Significantly different category^3^
A	3-Chlorobenzoate	4.7±1.4	B*, C**, D**, E**, F*
B	Fructose	2.2±0.4	A*, C*, D*, E*, F*
C	4-Hydroxybenzoate	0.6±0.2	A**, B*
D	Anthranilate	0.3±0.2	A**, B*, F*
E	Benzoate	0.1±0.3	A**, B*, C*
F	Glucose	0.7±0.5	A*, B*, D*

1 10 mM of carbon source in minimal medium (see Methods).

2 Average ICE*clc* transfer competent subpopulation of cells (percent of total) determined from biological triplicates, expressing *egfp* from P_int_ ± standard deviation. Sampled 15 - 20 h after onset of stationary phase. Determined via *R* command *find.sub.pop(…)* in *Default* mode.

3 * and ** indicate significant differences at P<0.05 and P<0.01, respectively, as determined by the Welch Two Sample t-test.

This method was termed *Default* in *R* to distinguish it from three other methods of subpopulation separation proposed in this study: *Manual*, *Boxplot1.5*, and *Boxplot3* (Protocol S1). *Manual* allows the user to manually distinguish large and small subpopulation by visually placing the cutoff value between the two subpopulations on a Q-Q plot (this can be done in *R* by use of the *locator(…)* function, which reads the position of the graphics cursor when the mouse button is pressed; see Protocol S2, Figure S3). Alternatively, the same procedure can also be carried out on a histogram, in which case the histogram peak-to-tail border has to be visually determined (Figure S3). Bates and collegues [42] deduced subpopulation size by determining the midpoints of histogram peaks. However, when comparing histogram mid-point determiation versus histogram peak-to-tail border determination as means to define subpopulatione we found the latter more precise (Figure S3). A similar idea based on manual placement of population separation aids has been used previously (although without the use of interactive graphs), where visually placed tangents in a CDF plot were employed and approximate reading by eye determined the cutoff point between small and large subpopulation [12]. The methods *Boxplot1.5* and *Boxplot3* both work simply by applying commonly used formulas for outlier detection in boxplots [58], [59]; here we consider the upper tail outliers as part of the small subpopulation and represent them as a percentage of the whole population. *Boxplot1.5* uses the formula 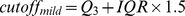, where Q_3_ is the 3rd quartile of the data, *IQR* the interquartile range, and *cutoff* the lower limit for mild outlier determination. Similarly, *Boxplot3* uses the formula 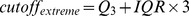 for extreme outlier determination.

### Method Comparison

**Figure 3:**
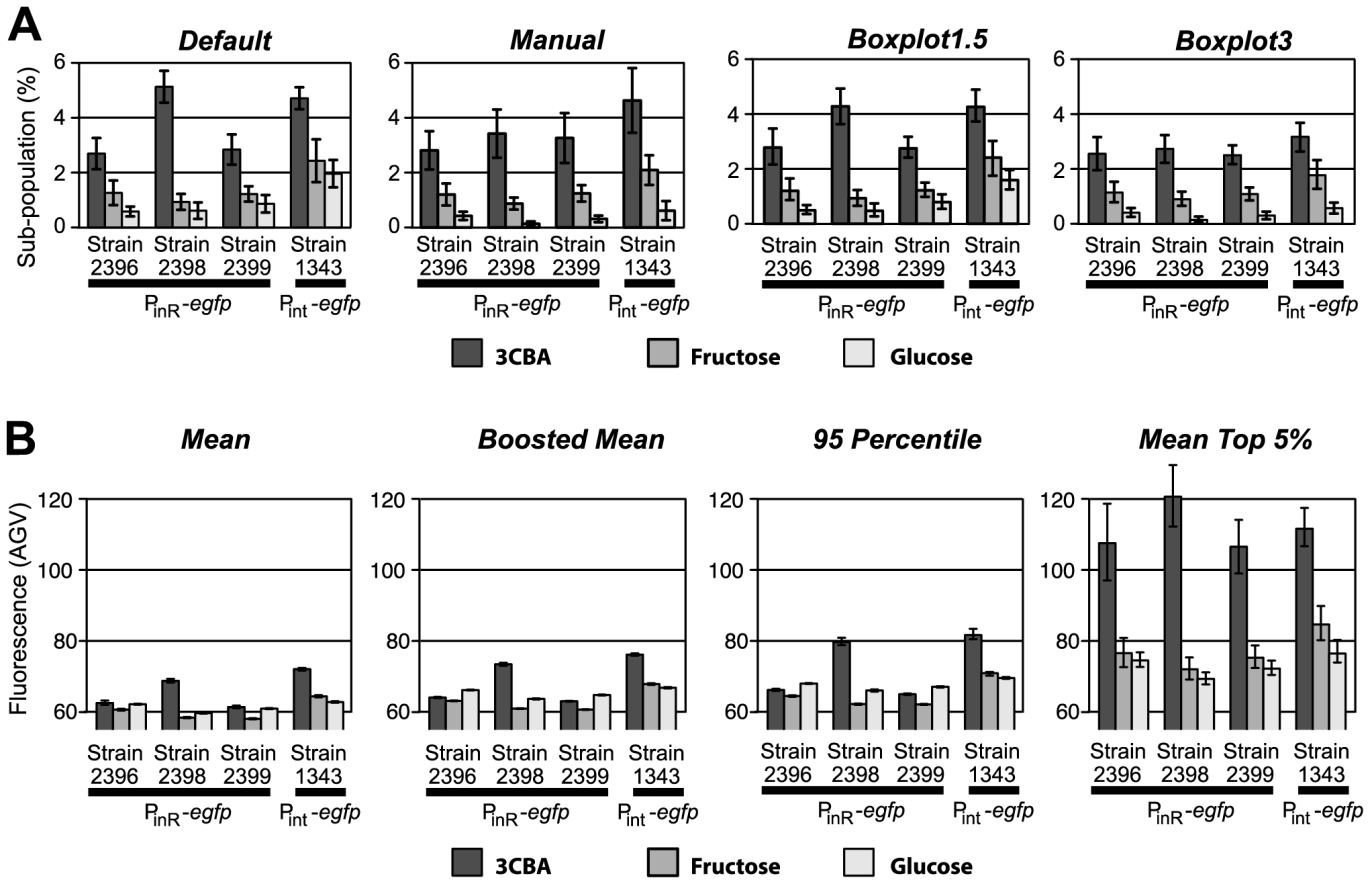
Different methods for quantification of subpopulation sizes of P_int_-*egfp* or P_inR_-*egfp* expressing cells. (A) Output of four different PS methods for subpopulation size. For each method the same data set was used. (B) Same data as (A) but quantified via distribution-independent non-PS methods that do not determine subpopulation size. Error bars indicate the 95% confidence interval for re-sampled (bootstrapped) data. Dark grey bars: 3CBA grown cells; intermediate grey bars: fructose-grown cells; light grey bars: glucose-grown cells.

To compare methods that relied on population splitting (PS) into large and small subpopulation (*Default*, *Manual*, *Boxplot1.5*, *Boxplot3*) to methods that did not (*Mean*, *Boosted Mean*, *95^th^ percentile*, *Mean Top 5%*), we analyzed small subpopulation sizes of cells defined by eGFP expression from both the P_int_ and the P_inR_ promoters inserted in single copy in *P. knackmussii* B13 derivatives, and grown under different conditions (Figure 3, Table S4, S5). *P. knackmussii* cultures in 3CBA were typically growing exponentially between 8 and 20 h after inoculation, whereas stationary phase (i.e., cessation of growth) was reached after 24 h (Table S2). *P. knackmussii* cultures in fructose were typically growing exponentially between 20 and 40 h after inoculation, and reached stationary phase after 45 h (Table S2). In contrast, *P. knackmussii* cultures on glucose grew slightly faster and reached stationary phase after 12 h (Table S2). We further tested benzoate, 4-hydroxybenzoate and anthranilate (Table 1). Cultures on anthranilate grew much slower, with stationary phase reached after 50 h (Table S2). Analysis of all culture conditions indicated that growth on 3CBA elicited the strongest induction of P_int_ and P_inR_ promoters in comparison to the others (Table 1, Figure 3, Table S4). Further, PS methods indicate that a larger subpopulation of P_int_-*egfp* expressing cells is formed on fructose in comparison to glucose, benzoate, and the other two aromatic compounds (Table 1, Figure 3A, Table S4). In contrast, with the exception of *Mean Top 5%*, non-PS methods failed to distinguish between 3CBA-, fructose- and glucose-grown induction (Figure 3B, Table S5). We therefore conclude that the PS methods are more sensitive to small but consistent changes in subpopulation sizes than non-PS methods.

### Method Robustness Analysis by Bootstrapping

**Figure 4:**
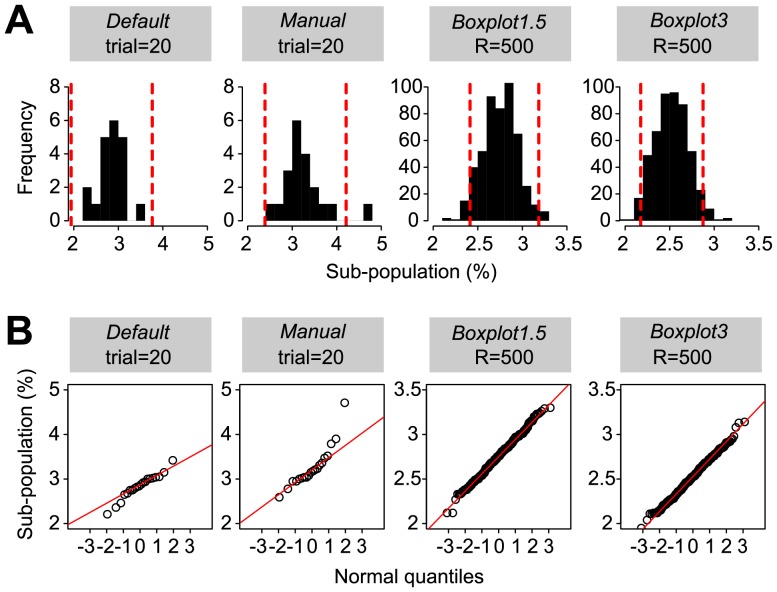
Effect of re-sampling methods of original data sets on the determination of confidence intervals for the subpopulation size of *egfp*-expressing cells in stationary phase cultures of *P. knackmussii* B13 strain 2399 (single copy P_inR_ *-egfp*) grown on 3CBA. (A) Bootstrapping of original data sets (re-sampling with replacement). Methods *Default* and *Manual* were repeated 20 times with manual intervention of the slope line determination. Methods *Boxplot1.5* and *Boxplot3* use 500 automatically re-sampled data sets. 95% confidence intervals (red, dotted lines) were calculated assuming a normal distribution of the results (mean±SD×1.96). (B) same data as in (A) but re-sampled subpopulation size determinations plotted as Q-Q plots. Note the normal distribution of the results.

**Figure 5:**
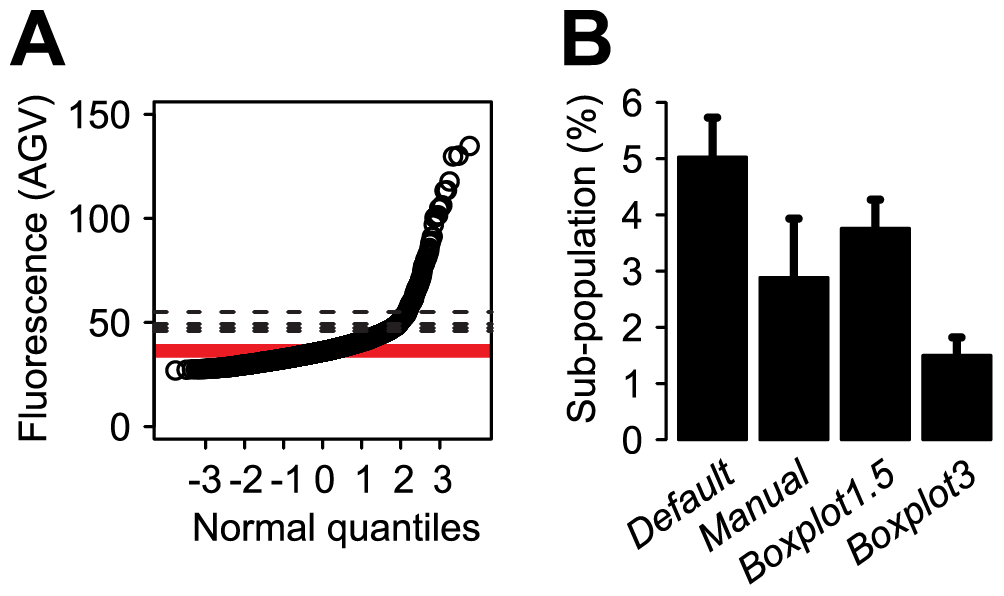
Example of a data set showing poorer Q-Q plot performance (smooth curve of distributed data points). (A) Q-Q plot of single cell eGFP fluorescence values obtained from *P. putida* UWC1-ICE*clc* P_int_-*egfp* cells (strain 2508) grown on 3CBA to early stationary phase. Width of the red line corresponds the interquartile range of the fluorescence values. Dotted lines indicate threshold line placement for subpopulation calculation via methods *Boxplot3*, *Boxplot1.5*, *Default* and *Manual*. (B) Results from the four different subroutines on this data set. Error bars represent 95% confidence intervals on re-sampled data sets with bootstrapping (see Figure 4).

In order to assess the robustness and accuracy of estimating small subpopulation sizes using different PS methods, we tested each PS method separately on a number of slightly varying bimodal populations. For this purpose we used bootstrapping with re-sampling (with replacement) data from wet experiments followed by the PS method and calculation of 95% confidence intervals. Bootstrapping was carried out with 20 replicates for the manual PS methods *Default* and *Manual*, and 500 replicates for all other methods, PS and non-PS. The bootstrapping procedures were implemented in the *R* functions *get.ci(…)* and *get.ci.other(…)* (Protocol S1) for PS and non-PS methods, respectively, both of which keep a record of the results after each replicate and calculate 95% confidence intervals (Figures 3, 4, 5). We compared eight different methods using the same data set including four PS (Figure 3A) and four non-PS methods (Figure 3B). Bootstrapping results indicate that, although less sensitive to small subpopulation changes, most non-PS methods are much more precise than PS methods; that is, they display smaller confidence intervals in response to random variations in data. An exception is the non-PS method *Mean Top 5%*, whose 95% confidence intervals look similar to those of the PS methods. Interestingly, *Mean Top 5%* is also the only non-PS method that confirmed a statistically significant eGFP fluorescence subpopulation change in *P. knackmussi* B13 P_int_ *-egfp*/P_inR_ *-egfp* grown on 3CBA versus grown on fructose or glucose (Table S4). However, *Mean Top 5%*, like all other non-PS methods but unlike most PS methods, failed to indicate a statistically significant difference between growth on fructose and growth on glucose (Table S5). The extreme robustness to random variation as seen in the methods *Mean*, *Boosted Mean*, and *95^th^ Percentile*, might explain part of the reason why these methods fail to respond significantly to small changes in small subpopulations (Figure 3B, Table S5). On the other hand, PS methods *Default*, *Manual*, *Boxplot1.5*, and *Boxplot3*, showed comparably large confidence intervals, reflecting some inconsistency in separating small subpopulations from large subpopulations (Figure 3A, Table S4). Nevertheless, all PS methods distinguished between small subpopulation sizes of 3CBA-grown versus fructose-grown or glucose-grown *P. knackmussii* B13 P_int_ *-egfp*/P_inR_ *-egfp*. Furthermore, PS methods *Manual*, *Boxplot1.5* and *Boxplot3.5* even showed significant differences between fructose-grown and glucose-grown *P. knackmussii* B13 P_int_ *-egfp*/P_inR_ *-egfp*. Thus, our experiments showed that, while non-PS methods are generally more robust to overall variation in populations, they are also less sensitive to small subpopulation changes than PS methods.

Confidence interval calculation via bootstrapping may be particularly useful in cases where subpopulation measurements are biased. As an example, subpopulation determination according to the PS method *Manual* is inherently biased due to human subjectivity in placing the cutoff point on a Q-Q plot where subpopulations should be separated. This problematic can be diminished, however, by repeating the method several times on a resampled dataset (bootstrapping) and calculating the confidence interval. As another example for the use of bootstrapping, normal Q-Q plot representation of P_int_ *-egfp* expression in *P. putida* UWC1 typically manifested as a curve (Figure 5) rather than the two lines of different slopes as seen in *P. knackmussii* B13 (Figure 2), which complicated the finding of the point of separation between subpopulations. However, re-applying PS methods on re-sampled datasets helped to define the confidence limits of the subpopulation determination itself (Figure 5). Another demonstration of such a case is shown in Figure S1, where a dataset that includes biases due to faulty data recording during image acquisitions is subjected to PS methods. This case also highlights the usefulness of summarizing single cell data as boxplots per image, which makes it possible to filter out image-inherent bias in a data set (Protocol S2).

### Computer Simulations

**Figure 6:**
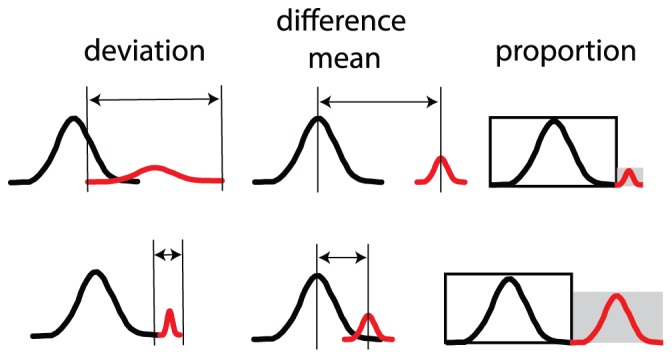
Scheme illustrating the three parameters, mean difference between large and small subpopulation, standard deviation of small subpopulation and proportion of small subpopulation, that were changed in a computer simulation to create variations of mixed populations upon which the PS methods of subpopulation determination were tested (see Figure 7, 9). Black, large subpopulation. Colour, small subpopulation.

**Figure 7:**
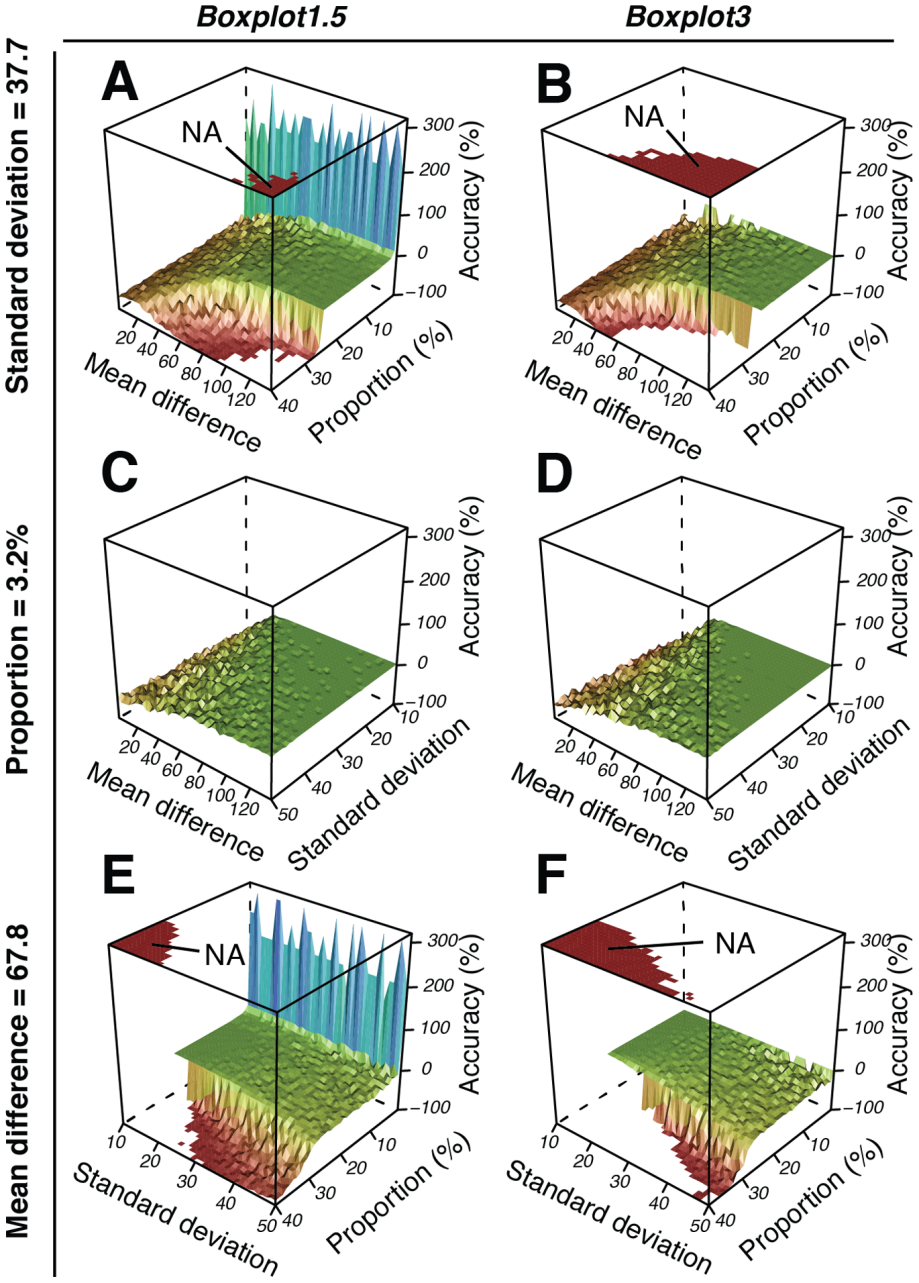
3D surfaces of simulation data showing the accuracy (*z* axis) in the estimated compared to the true subpopulation size using two different methods of population separation: *Boxplot1.5* and *Boxplot3*. Accuracy is shown as a function of different population mixtures (1600 per plot), with subpopulations either varying in mean differences (range: 2–137; n = 40; *x* axis) and proportions (range: 0.1–40%; n = 40; *y* axis) at a constant standard deviation (37.7) (A, B), or varying in mean differences (range: 2–137; n = 40; *x* axis) and standard deviations (range: 10–50; *y* axis) at a constant proportion (3.2%) (C, D), or with varying standard deviations (range: 10–50; n = 40; *x* axis) and proportions (range: 0.1–40%; n = 40; *y* axis) at a constant mean difference (67.8) (E, F). Accuracy is expressed as the percent difference between calculated and real subpopulation size, and therefore indicates the normalized deviance of the calculated subpopulation size from the real subpopulation size. A negative value indicates that the method underestimated the subpopulation size. A positive value indicates an overestimated result. A value of zero indicates absolute accuracy. A smooth surface of the same colour/grey-level indicates a robust separation. NA, missing values.

**Figure 8:**
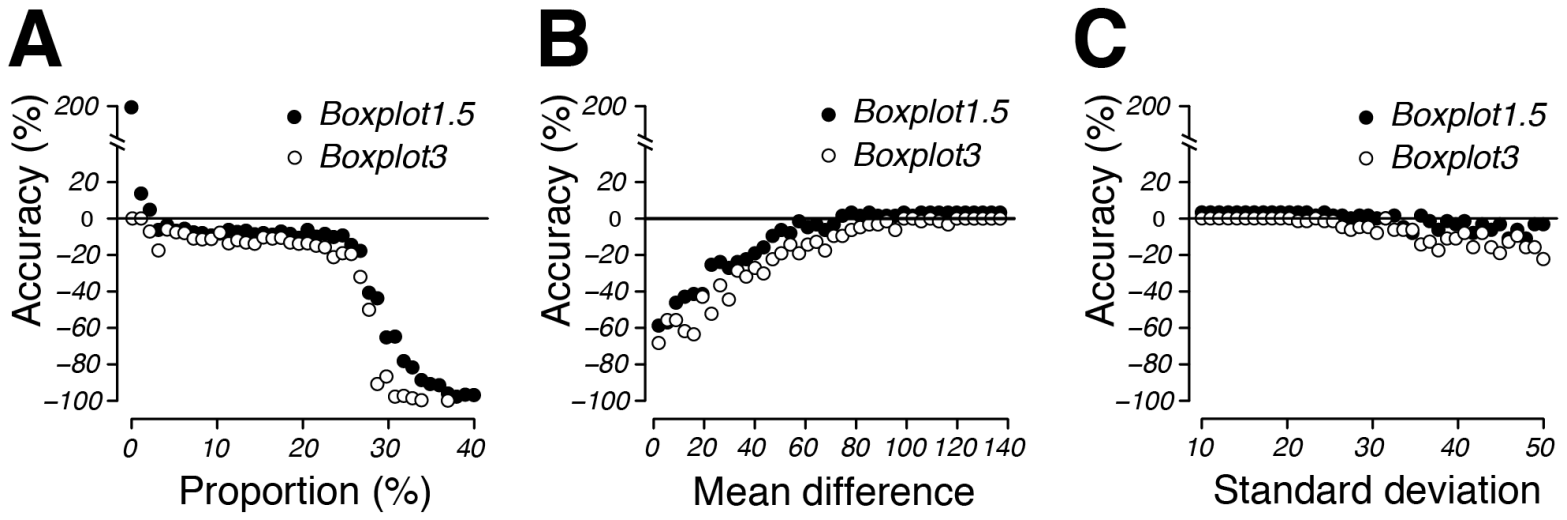
2D representations of simulations shown in Figures 7A–F. Accuracy is shown as a function of subpopulation proportion (range: 0.1–40%; n = 40) at a mean difference of 67.8 and a subpopulation standard deviation of 37.7 (A), as a function of mean difference (range: 2–137; n = 40) at a subpopulation proportion of 3.2% and subpopulation standard deviation of 37.7 (B), or as a function of subpopulation standard deviation (range: 10–50; n = 40) at a mean difference of 67.8 and a subpopulation proportion of 3.2% (C). Also see Table S6 for values of these graphs.

Following the empirical bootstrap approach above, we wanted to test the performance of our proposed methods on a large variety of bimodal populations. Hereto we used computer simulations that not only allowed to treat large data sets but also had the added advantage that true subpopulation parameters were known before analysis. Thus, by comparing true and estimated subpopulation ratios, the accuracy (in percent) of each PS method in estimating subpopulation proportions could be assessed, which we calculated according to: 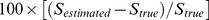, where *S_estimated_* is the estimated subpopulation size, and *S_true_* is the true subpopulation size, both expressed as a percentage of the total population. In this way we could consider how the accuracy varies with different bimodal population parameters. In a first experiment we tested the accuracy of separating two subpopulations across a range of 64,000 simulated bimodal populations (Figure 6, 7, Video S1–S3). The populations were produced by mixing a single large subpopulation with a variety of smaller-sized subpopulations (Figure 6). To create the large subpopulation we used typical population parameters as found in non-induced populations of *P. knackmussii* B13 containing a P_inR_-*egfp* fusion (Table S3). By plotting the calculated accuracy against true subpopulation size, true subpopulation standard deviation and/or true subpopulation mean, we now obtained an overview of the accuracy and robustness of the separation method, presented, for space reasons, either as selected representative 3D plots (Figure 7) or selected representative 2D plots (Figure 8). However, the complete data set can be viewed in 4D as movies (Video S1–S3). Only two of the four separation methods were tested in this way, *Boxplot1.5* and *Boxplot3*, since it would have been an almost impossible feat to test the other methods *Default* and *Manual* on an equally large number of datasets due to their requirement of a manual work-flow (mouse-clicking on an interactive graph). However, *Default* and *Manual* were still tested on a smaller scale including fewer simulated bimodal populations (Table 2). The simulation results show that *Boxplot1.5* and *Boxplot3* produce estimates within 20% of the true value over the entire span of tested subpopulation standard deviations (10–50) as long as the difference between means of the large and the small populations remains between 40 and 50 units, respectively (Figure 7, Figure 8, Video S1–S3, Table S6). Furthermore, the simulations indicated that subpopulation size estimation becomes less accurate when its size is decreasing to below 1.1% or values in the small subpopulation become more diverse (i.e., higher standard deviation) (Figure 7, 8, Video S1–S3, Table S6). Both methods also become rapidly unreliable when small subpopulation proportions become larger than 25% (Figure 7, 8, Video S1–S3, Table S6), a feature also confirmed in another simulation experiment (Table 2). This is because outlier detection in boxplots beyond this point is not synonymous with bimodality anymore (Figure S2). However, we found that the Q-Q plot-based PS methods *Manual* and *Default* could still be used to accurately determine subpopulations larger than 25%, since Q-Q plots show bimodality over a large range of subpopulation proportions (Table 2, Figure S2).

**Table 2:** Comparison of estimated to true subpopulation sizes in simulated bimodal populations by using different separation methods.

True subpopulationsize (% of total population)^1^	Estimated subpopulation size (% of total population)^2^			
	*Default*	*Manual*	*Boxplot1.5*	*Boxplot3*
1.00	1.75±0.17	0.98±0.03	1.17±0.18	0.98±0.06
3.00	3.80±0.93	2.77±0.20	2.92±0.08	2.63±0.21
6.00	5.80±0.31	5.85±0.26	5.57±0.03	5.45±0.05
9.00	8.80±0.10	8.40±0.30	8.28±0.13	7.75±0.35
12.00	11.35±0.15	11.58±0.35	10.97±0.12	10.42±0.13
15.00	14.25±0.18	14.12±0.13	13.85±0.10	13.15±0.33
18.00	17.02±0.18	16.67±0.28	16.52±0.25	15.43±0.28
20.00	18.88±0.13	18.73±0.45	17.83±0.06	16.80±0.26
30.00	27.50±0.23	28.58±0.38	12.47±2.32	2.42±1.06
40.00	37.13±0.56	38.05±0.26	1.93±0.31	0.03±0.03
50.00	46.02±0.30	46.18±0.60	0.30±0.15	0.03±0.03
60.00	54.32±0.38	56.95±0.28	0.10±0.10	0.02±0.03
70.00	63.02±0.49	66.02±0.21	0.03±0.03	0.03±0.03
80.00	70.88±1.16	74.55±0.17	0.05±0.00	0.03±0.03
90.00	75.40±2.16	84.32±1.08	0.17±0.14	0.03±0.03
92.00	75.64±1.58	87.01±0.38	0.25±0.15	0.00±0.00
95.00	71.42±1.00	90.03±0.58	0.35±0.17	0.03±0.03
98.00	50.59±18.55	92.20±0.10	0.32±0.18	0.03±0.03

1 True subpopulations were simulated using the *R* function *rnorm(…)* with a standard deviation of 37.7, a mean value of 127.3, and the number of observations corresponding to the subpopulation percentage to be tested from a total number of 2000 observations. Mean and standard deviation used for the simulations represent population parameters as obtained from fluorescence microscopy analysis of batch grown *P. knackmussii* B13 P_int_ *-egfp* in 3CBA (see Table S3).

2 Estimated subpopulation sizes (mean ± SD; 3 independent repetitions) were determined applying the PS methods on simulated bimodal populations using the *R* function *findsub(…)* (Protocol S2). A bimodal population was simulated by mixing two simulated populations, a real subpopulation^1^ and a second subpopulation. The second subpopulation was created using the *R* function *rnorm(…)* with a standard deviation of 3.9, a mean value of 63.0, and the number of observations depending on the sample size of real subpopulation^1^ to give a total of 2000 observations. Mean and standard deviation used for the simulations represent population parameters as obtained from fluorescence microscopy analysis of batch grown *P. knackmussii* B13 P_int_ *-egfp* in 3CBA (see Table S3).

**Figure 9:**
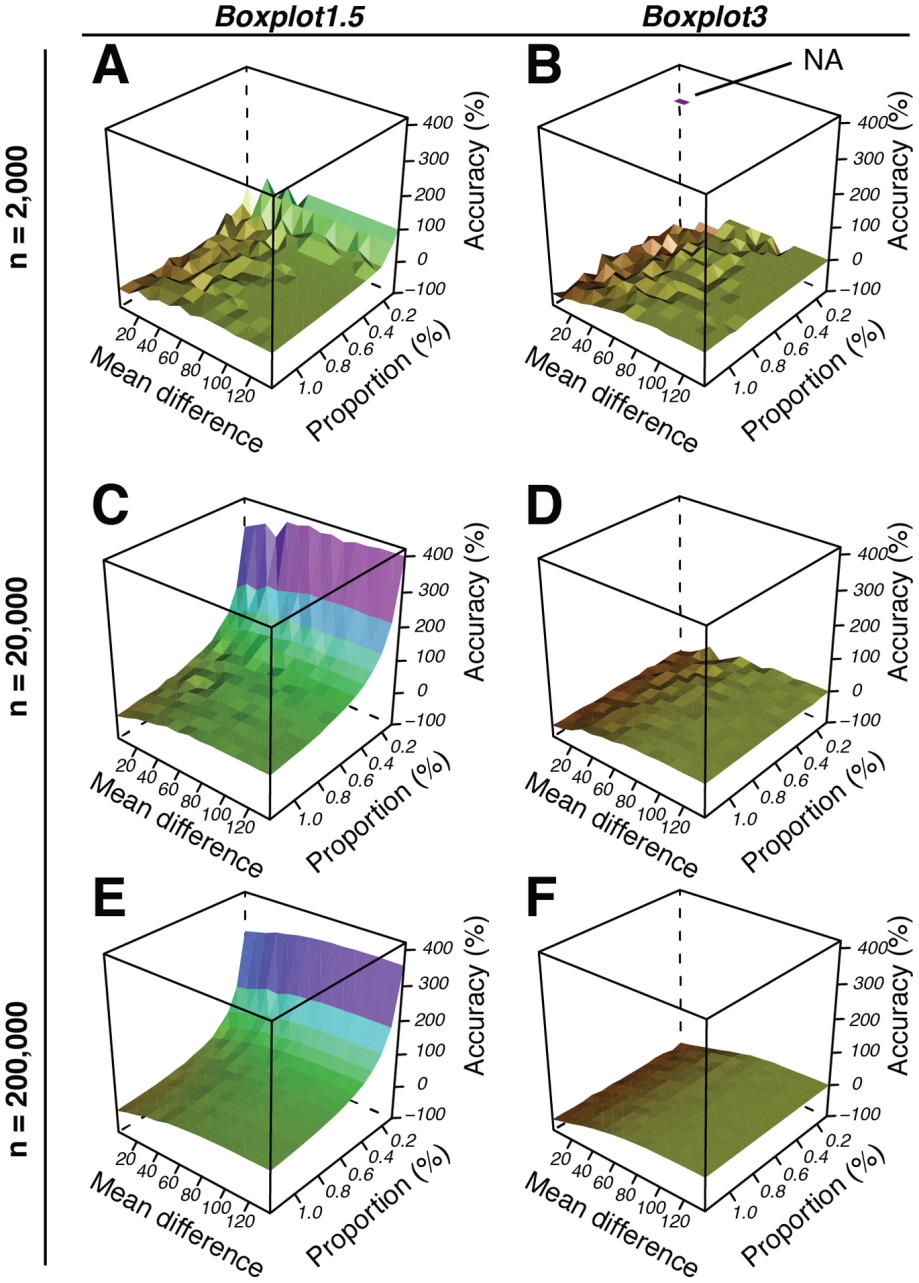
Same as Figure 7 but as a result from a simulation focusing on subpopulations with small proportions (range: 0.1–1.2%). Each surface is constructed from 250 data points, stemming from population separations of population mixtures with varying subpopulations with 15 different mean difference values (range: 2–137) and 15 different proportion values (range: 0.1–1.2%) at a constant standard deviation of 38.6. (A, B): Simulation was performed with population mixtures with n = 2000. (C, D): Simulation was performed with population mixtures with n = 20,000. (E, F) Simulation was performed with population mixtures with n = 200,000.

**Figure 10:**
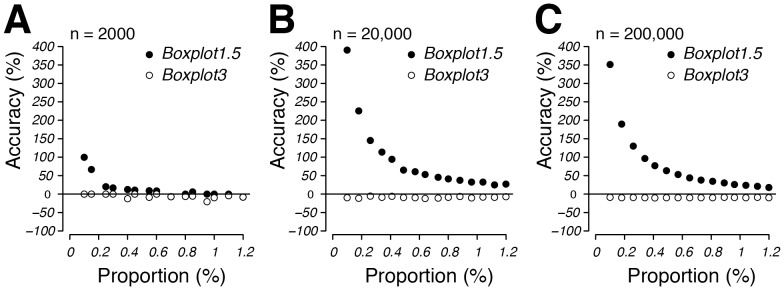
2D representations of simulations shown in Figures 9A–F. Accuracy is shown as a function of subpopulation proportion (range: 0.1–1.2%; n = 15) at a mean difference of 67.8 and a subpopulation standard deviation of 37.7. (A): Simulation was performed with population mixtures with n = 2000. (B): Simulation was performed with population mixtures with n = 20,000. (C): Simulation was performed with population mixtures with n = 200,000. Also see Table S7 for values of these graphs.

With respect to the decreasing accuracy with decreasing small subpopulation sizes we conducted a second series of simulations dedicated to very small subpopulation sizes focussing on subpopulation proportions between 0.1 and 1.2% (Figure 9, 10, Video S4–S9, Table S7). Overall, *Boxplot3* manifested itself as the more precise and accurate method than *Boxplot1.5* for determining very small subpopulations. More specifically, *Boxplot3* estimates were never more than 11% inaccurate from the true value (n = 200000) over the entire span of percentage parameters tested, provided the mean difference was at least 67.8 units and standard deviation was set at 37.7 units (Figure 10, Table S7). By comparison, under the same conditions, *Boxplot1.5* estimates were within 20% accuracy of the true value only when the tested subpopulation was larger than 1.1%, exponentially increasing to 352% where subpopulations were approaching 0.1% (Figure 10, Table S7).

## Discussion

### Principal Contribution of the Study

The principal contribution of this study is a simple and practical statistical approximation to subpopulation quantification in bimodal populations. For this purpose we created a set of functions in the open source software environment *R* accompanied by a step-by-step instructional protocol for easy implementation (Protocol S1, 4).

### Motivation of this Study

The motivation to define methods of subpopulation quantification was twofold: firstly stemming from a need for a statistical tool do describe subpopulation sizes of ICE*clc* transfer competent cells in *Pseudomonas* in particular [12], [13], [60] and, secondly, to provide a more general set of tools for basic subpopulation quantification in single cell microbiology with easy implementation into existing image analysis work-flows.

### Why Try to Distinguish between Subpopulations?

Population-level parameters, such as the average cellular response, by definition will obscure biological detail that is noticeable in small subpopulations of cells. The task of determining the subpopulation sizes of ICE*clc*-transfer competent cells in *P. knackmussii B13* presents itself as a particularly challenging example. Firstly, this is because their proportions are typically small (3.3% of the total population; see Table S3) [11], [13]; secondly, they commonly have an estimated mean expression value from the key P_int_-promoter that is only twice as high as the mean of the non-active population (Table S3). Thirdly, the standard deviation of expression values in this subpopulation is ca. 10 times larger than that of the non-active population (Table S3). Together, this equates to subpopulations that are almost certainly overlapping and thus mixed to some degree, which makes it mathematically impossible to achieve “true” demarcation between subpopulations [46]. Histograms of ICE*clc*-activity distributions typically resemble Gaussian curves with hardly noticeable tails extending to their right-hand sides (Figure 1). First, we speculated that such histograms are unsuitable visualisations for manually placing subpopulation thresholds confidently and in a statistically acceptable way; even if a threshold was placed such that the histogram tail would be separated from the Gaussian curve, we questioned the reproducibility of such a placement due to a manual work-flow based on grounds of visual perception. Such an approach, we assumed, was likely be prone to bias (user arbitrariness) by subjective decision-making, therefore hindering reliable quantification of subpopulation changes. Indeed, Bates and collegues [42], [43] offered a “manual” histogram-based approach earlier, which we noticed produced strong variability (imprecision) in subpopulation size determination of ICE*clc* transfer competent cells (Figure S3). Hence, we decided to improve upon this by using Q-Q plot representations. These have the added advantage of showing two subpopulations, each with normally distributed data of different spread, as two straight lines of different slopes (see, e.g., Figure 2) [30]. The point of demarcation between such subpopulations can be determined manually (as in the subroutine *Manual*). Yet, in cases where bimodal distribution patterns are less clear (e.g., Figure 5), we developed a method (named *Default*) that standardizes cutoff placement on grounds of the most reproducible part of the distribution pattern, that is, the part that is most robust to change by subpopulation effects. In a Q-Q plot this region conveniently corresponds to the lower (and longer) straight line, on which an interval of representative slope of that line should be easily definable. Nevertheless, under certain conditions *Manual* can be the more accurate tool (Table 2, Figure S3) and is especially useful in cases where the *Default* algorithm fails, for example in instances with datasets where the IQR of the larger subpopulation does not follow a Gaussian distribution (Figure S1). Generally, when subpopulation quantification becomes challenging and ambiguous, or risks to be influenced by subjective input from the user, it is good practice to apply quantification repeatedly on the same original but re-sampled (with replacement) data set. Importantly, both *Default* and *Manual* are not limited by the proportions of the tester subpopulation in order to produce quantitatively correct results (Table 2), in contrast to *Boxplot* methods.

*Boxplot1.5* and *Boxplot3* define subpopulations without prompting the user for input since their subpopulation classification is simply based on outlier detection as commonly used in boxplots [54], [58], [59]. This latter trait was found especially useful when numerous data sets needed to be analysed as shown in the simulations of this study, where 64,000 bimodal populations were analysed within ca. 10 hours (see Figure 7, 9). As expected, Boxplot methods respond differently than manual methods to changes in distribution patterns (Figure 5, Figure S1, S2). *Boxplot1.5* and *Boxplot3* only allowed for accurate subpopulation quantification where these amounted to less than 25% of the total population (Figure 7, 8, Table 2, Figure S2). This is not surprising, since it is well documented that under certain distribution scenarios boxplots fail to visualize bimodality [61]. *Boxplot3* was by far more accurate than *Boxplot1.5* with subpopulations smaller than 1% of the total population (Figure 9, 10, Video S4–S9, Table S7). In contrast, due to its more conservative classification of outliers, *Boxplot3* tends to underestimate subpopulation sizes in comparison to *Boxplot1.5* (Figure 8). At low subpopulation sizes, it is relevant to increase sample numbers such as can be obtained from flow cytometry experiments (>20000; see Figure 9), or calculate confidence limits from bootstrappings (e.g., function *get.ci(…),* Figure 5, Protocol S2). In general, when comparing PS methods to existing methods of quantification, we found that they were more sensitive to small subpopulation effects, for example when assessing ICE*clc* activity in *P. knackmusssii* B13 under different growth conditions (Figure 3, Table S4).

### Limitations of the Proposed Methods

The strength of the proposed methods in this paper is also their weakness; the determination of the percentile corresponding to the cutoff point or threshold between two subpopulations can only be approximated, and becomes more inaccurate as subpopulations overlap. On the one hand the approximation allows to split a bimodal population into two and characterize the biologically relevant fraction in a subpopulation response. On the other hand such characterization becomes increasingly inaccurate in describing the biologically relevant fractions until it eventually fails completely as subpopulations overlap. There are only two alternatives to this dilemma, which are analyses that either avoid finding subpopulation-relevant cut-off percentiles altogether, or describe all percentiles in a population, without specifying one. An example of the second approach are visualisations of qualitative changes of entire populations through comparisons of distribution patterns [30], . MacArthur [46] even proposed a way to quantify qualitative changes spanning the total range of percentiles, that is calculating percentage differences per individual percentile between treatment and control (Figure S4, Table S8) [46].

### Other Studies

Few studies in the microbiology literature specify the problematic of statistically exposing true subpopulations from bimodal populations. Rather, it seems that most studies content themselves with a categorisation of subpopulations via thresholds based on fluorescence background levels, negative controls lacking fluorescent marker, or manual gating of clusters in flow cytometry [11], [17], [21], [26], [33], [36]–[38], [47]. The reasons might be twofold. Firstly, pragmatism, which argues that as long as an approach serves the purpose of quantification at a sufficiently high resolution it is good enough. Secondly, the problematic that statistical distributions of subpopulation behaviours overlap, causing a certain degree of subpopulation mixing, and therefore make a precise demarcation between subpopulations impossible.

### Conclusion

To date there exists no universal protocol in the microbiology literature for the determination of small subpopulation sizes. Rather, many labs use their own in-house methods of subpopulation quantification. We see the advantage and novelty of our proposed methods in the attempt to statistically deduce subpopulation size from a qualitative assessment of the underlying bimodal distributional shape. We argue that a distribution shape-based approach is by definition (inherently) more accurate in determining the true biologically relevant subpopulation than distribution-independent methods. Consequently, our approach should help firstly, to minimize inconsistencies in subpopulation classification caused by manual threshold placements, and secondly, to increase sensitivity and accuracy to subpopulation changes. Thirdly, our method would help to standardize subpopulation evaluation across different experimental set-ups. Since subpopulation size as expressed as percentage of the total population is a dimension-less quantity, it is also independent of scales and units linked to the sensitivity of recording equipments and experimental set-ups. Therefore, subpopulation size expressed as a fraction of the total population represents a suitable parameter for comparisons across a wide range of different studies.

## Supporting Information

Figure S1 **Bias compromises detection of small subpopulations in bimodal data.** This file contains a series of graphs that demonstrate the obstructive role of bias in estimating subpopulation size in bimodal data. The left row of graphs are based on a faulty data set with data originating from two images that have much lower fluorescence values as a result of a mistake during image acquisition. The right row of graphs represents the same data set but with the data from the biased images removed. This panel of graphs highlights the practicality of summarizing single cell data as boxplots per image, which makes it possible to find the source of bias in a data set.
(PDF)

Figure S2 **Failure of the method** ***Boxplot1.5*** **and success of the method** ***Default*** **to accurately analyze a bimodal population that contains a large subpopulation (40% of the total population).** In this file the failure of the method *Boxplot1.5* and the success of the method *Default* to accurately analyze a simulated bimodal population that contains a large subpopulation (40% of the total population) is demonstrated.
(PDF)

Figure S3 **Accuracy of a hand-analysis method estimating small subpopulation sizes in simulated bimodal populations via mid-point determination of large subpopulation histogram peak.** This file contains a graphical explanation of a hand-analysis method for subpopulation detection which uses visual determination of the mid-point of the large subpopulation peak in a histogram as a basis. A similar hand-analysis method has been proposed recently by Bates and collegues [42]. Further, this file contains a data-table showing the accuracy performance of the method on multiple simulated bimodal populations, and an annotated script which was used for the simulations in *R*.
(PDF)

Figure S4 **The use of P-P plots for non-parametric and graphical response quantification.** This file illustrates the concept of employing P-P plots for non-parametric and graphical response quantification [46], using results obtained from the measurement of ICE*clc* activity in *P. knackmussii* B13 grown under different environmental conditions as an example data-set.
(PDF)

Table S1 **Bacterial strains used in this work. In this file we provide a list with all bacterial strains used in this work.**
(DOC)

Table S2 ***Pseudomonas knackmussii*** **B13 growth in batch culture.** This file contains a table listing timing of exponential growth and onset of stationary phase in batch cultures of *P. knackmussii* B13 and *P. putida* UWC (ICE*clc*) grown on different carbon substrates.
(DOC)

Table S3 **Large and small subpopulation parameters of fluorescence data from promoter-** ***egfp*** **reporters for ICE** ***clc*** **activation in** ***Pseudomonas knackmussii*** **B13.** This file contains a data table showing typical measured large and small subpopulation parameters of fluorescence data obtained from promoter-*egfp* reporters for ICE*clc* activation in *P. knackmussii* B13 after growth on 3CBA. These parameters were used as reference parameters for ICE*clc* activation when creating some of the simulated subpopulations in Figures 7, 8, 9 and 10, Table 2, and Figure S2, S3.
(DOC)

Table S4 **Significance testing of subpopulation effects from ICE** ***clc*** **activation under different conditions quantified by different PS methods.** This file contains a data table showing results from quantifications of small subpopulation effects by different PS methods. Results from this table are visualized in Figure 3A.
(DOC)

Table S5 **Significance testing of subpopulation effects from ICE** ***clc*** **activation under different conditions quantified by different non-PS methods.** This file contains a data table showing results from quantifications of small subpopulation effects by different PS methods. Results from this table are visualized in Figure 3B.
(DOC)

Table S6 Accuracy as a function of subpopulation proportion (range: 0.1–40%; n = 40) at a mean difference of 67.8 and a subpopulation standard deviation of 37.7. This file contains a data table showing numerical data corresponding to Figure 8.
(DOC)

Table S7 **Accuracy as function of subpopulation proportion (range: 0.1–1.2%; n = 15) at a mean difference of 67.8 and a subpopulation standard deviation of 37.7.** Data table corresponding to Figure 10.
(DOC)

Table S8 **ICE** ***clc*** **activity-response in** ***Pseudomonas knackmussi*** **B13 P_int_-** ***egfp*** **to pre-growth on different carbon sources, quantified over percentile range.** Data correspond to Figure S4C.
(DOC)

Protocol S1 **Description of** ***R*** **functions for quantification of low abundance phenomena in bimodal populations.** This file provides a detailed description of the proposed *R* functions *findsub(…)* and *get.ci(…)* as tools for quantification of small subpopulation phenomena and method confidence interval calculation, respectively. We also show examples of graphical and command-line output from these functions.
(PDF)

Protocol S2 **Scripts and functions for quantification of low abundance phenomena in bimodal populations.** This file contains the proposed *R* scripts and functions for quantifying low abundance phenomena in bimodal populations. Comments within scripts and the README file serve as step-by-step guidance for the implementation of the relevant functions in *R*. An example data set is included, allowing for a demonstration of the relevant functions while following the step-by-step procedure.
(ZIP)

Protocol S3 **Scripts and functions for generating simulated data.** This file contains the *R* scripts and functions used for generating the simulated bimodal populations that were analyzed in this paper.
(ZIP)

Video S1 **Accuracy of subpopulation determination as quantified by the methods** ***Boxplot1.5*** **or** ***Boxplot3*** **from different simulated bimodal populations.** This file contains a movie showing the results of *Boxplot1.5* and *Boxplot3* methods of subpopulation detection tested on simulated bimodal populations with varying subpopulation proportions, standard deviations and set mean difference of 137 (see Methods). Method accuracy is shown as the percentage between estimated and true subpopulation size (z-axis), and as a function of subpopulation standard deviation (x-axis) and subpopulation proportion (y-axis). The 40 different movie image frames show results for different simulated subpopulation mean values. A value of zero indicates that estimated subpopulation size equals true subpopulation size. Negative or positive values indicate under- or over-estimation of subpopulation size in comparison to true subpopulation size, respectively. Instances where the method fails to detect any subpopulation size are indicated as solidly coloured squares at the top surface of the co-ordinate system (also see NA annotations in Figure 7, 9).
(MOV)

Video S2 **As Video S1 but with set standard deviation of 10.**
(MOV)

Video S3 **As Video S1 but with set subpopulation proportion of 40%.**
(MOV)

Video S4 ***Boxplot1.5*** **method accuracy as tested on simulated bimodal populations with low subpopulation proportions (0.1–1.2%).** This file contains a movie showing the results of the *Boxplot1.5* method of subpopulation detection tested on simulated bimodal populations of three different population sizes (n = 2×10^3^, n = 2×10^4^, and n = 2×10^5^) with simulated small subpopulation proportions ranging between 0.1 and 1.2% of the large subpopulation (see Methods). Set mean difference = 2.
(MOV)

Video S5 **As Video S4 but with set subpopulation proportion of 0.1%.**
(MOV)

Video S6 **As Video S4 but with set standard deviation of 10.**
(MOV)

Video S7 ***Boxplot3*** **method accuracy as tested on simulated bimodal populations with low subpopulation proportions (0.1–1.2%).** This file contains a movie showing the results of the *Boxplot3* method of subpopulation detection tested on simulated bimodal populations of three different population sizes (n = 2×10^3^, n = 2×10^4^, and n = 2×10^5^) with simulated small subpopulation proportions ranging between 0.1 and 1.2% of the large subpopulation (see Methods). Set mean difference = 2.
(MOV)

Video S8 **As Video S7 but with set subpopulation proportion of 0.1%.**
(MOV)

Video S9 **As Video S7 but with set standard deviation = 10.**
(MOV)
