# Supplementary material for: Improved Statistical Analysis of Low Abundance Phenomena in Bimodal Bacterial Populations
Source: PLoS One. 2013 Oct 30;8(10):e78288. doi: 10.1371/journal.pone.0078288 (PMC3813492; doi:10.1371/journal.pone.0078288)
Supplement: Figure S1 — Bias compromises detection of small subpopulations in bimodal data. This file contains a series of graphs that demonstrate the obstructive role of bias in estimating subpopulation size in bimodal data. The left row of graphs are based on a faulty data set with data originating from two images that have much lower fluorescence values as a result of a mistake during image acquisition. The right row of graphs represents the same data set but with the data from the biased images removed. This panel of graphs highlights the practicality of summarizing single cell data as boxplots per image, which makes it possible to find the source of bias in a data set. (PDF) [file pone.0078288.s001.pdf]

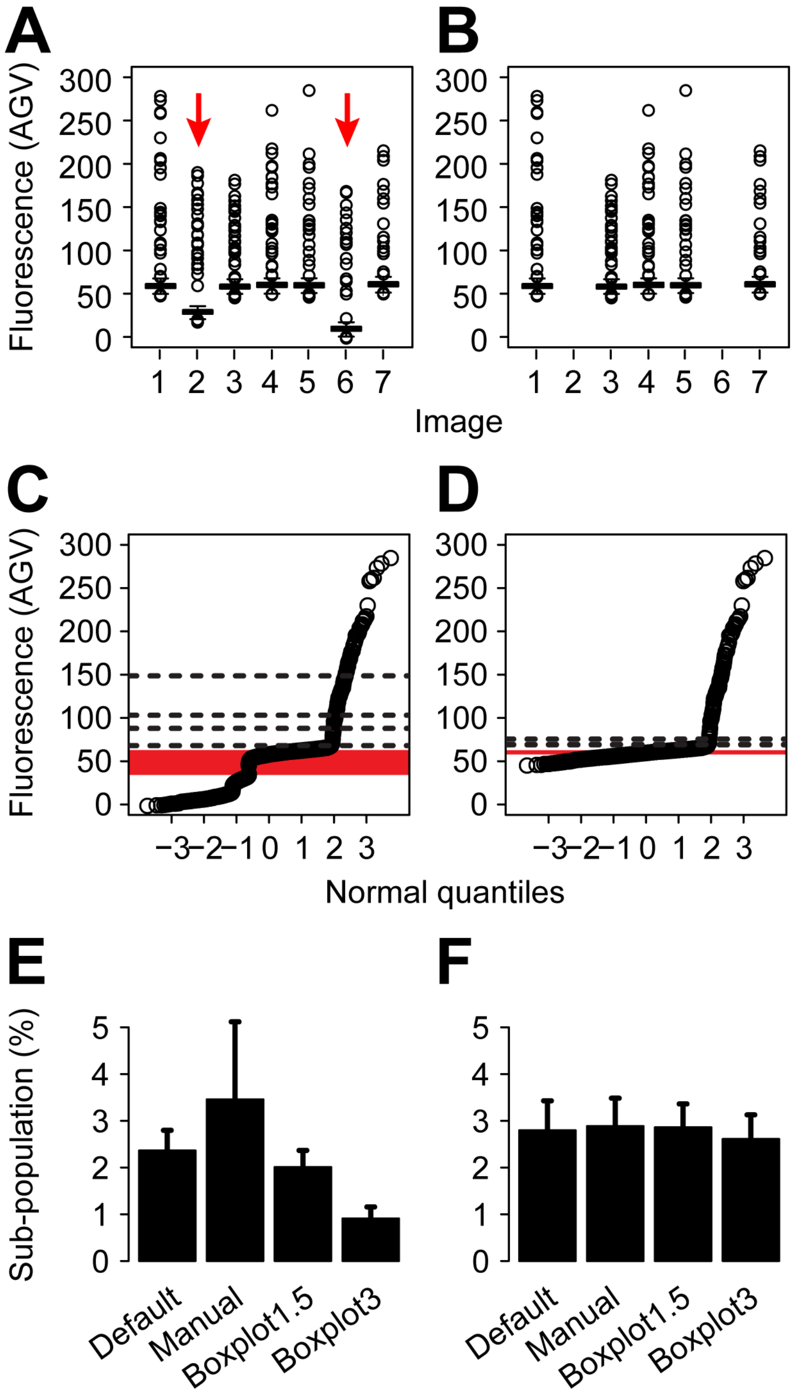

Figure S1 Bias compromises detection of small subpopulations in bimodal data.

This file contains a series of graphs that demonstrate the obstructive role of bias in estimating subpopulation size in bimodal data. A), B) Box-plot representations of P_{int}-*egfp* expression in *P. putida* UWC1 for 7 different image sets (1-7). The left row of graphs are based on a faulty data set with data originating from two images that have

much lower fluorescence values as a result of a mistake during image acquisition. The right row of graphs represents the same data set but with the data from the biased images removed. C), D) Q-Q plot representations of uncorrected and corrected data sets in A and B, respectively. E), F) Subpopulation size calculations using the uncorrected and corrected data sets from A and B, respectively. This panel of graphs highlights the practicality of summarizing single cell data as boxplots per image, which makes it possible to find the source of bias in a data set.
