# Supplementary material for: Improved Statistical Analysis of Low Abundance Phenomena in Bimodal Bacterial Populations
Source: PLoS One. 2013 Oct 30;8(10):e78288. doi: 10.1371/journal.pone.0078288 (PMC3813492; doi:10.1371/journal.pone.0078288)
Supplement: Figure S2 — Failure of the method Boxplot1.5 and success of the method Default to accurately analyze a bimodal population that contains a large subpopulation (40% of the total population). In this file the failure of the method Boxplot1.5 and the success of the method Default to accurately analyze a simulated bimodal population that contains a large subpopulation (40% of the total population) is demonstrated. (PDF) [file pone.0078288.s002.pdf]

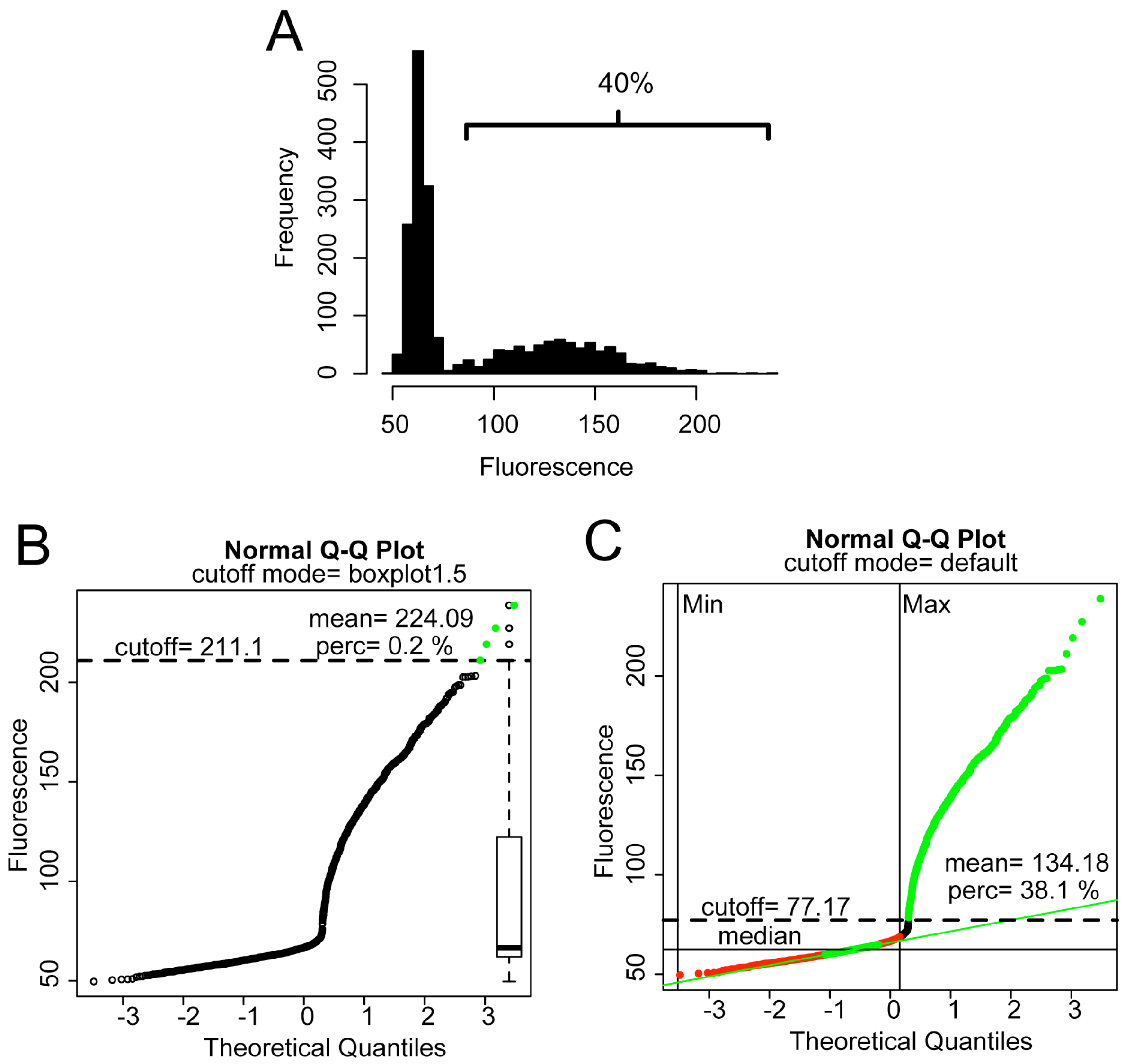

Figure S2. Failure of the method *Boxplot1.5* (B) and success of the method *Default* (C) to accurately analyze a simulated bimodal population (A; n=2000) containing a large subpopulation (40% of the total population). Bimodal population was created in *R* by mixing two simulated Gaussian subpopulations whose means (63.0, 127.3) and standard deviations (3.9, 37.7) represented typical population parameters as obtained from fluorescence microscopy analysis of batch grown *P.knackmussii* P_{int}-*egfp* in 3CBA (see Additional file 5). mean, estimated mean of subpopulation; perc, estimated percentage of subpopulation from total population; cutoff, point of separation between subpopulations; Min, estimated minimum of lower subpopulation; Max, estimated maximum of lower subpopulation; median, estimated median of lower subpopulation; Green points above cutoff, estimated subpopulation.
