# Supplementary material for: Improved Statistical Analysis of Low Abundance Phenomena in Bimodal Bacterial Populations
Source: PLoS One. 2013 Oct 30;8(10):e78288. doi: 10.1371/journal.pone.0078288 (PMC3813492; doi:10.1371/journal.pone.0078288)
Supplement: Figure S3 — Accuracy of a hand-analysis method estimating small subpopulation sizes in simulated bimodal populations via mid-point determination of large subpopulation histogram peak. This file contains a graphical explanation of a hand-analysis method for subpopulation detection which uses visual determination of the mid-point of the large subpopulation peak in a histogram as a basis. A similar hand-analysis method has been proposed recently by Bates and collegues [42]. Further, this file contains a data-table showing the accuracy performance of the method on multiple simulated bimodal populations, and an annotated script which was used for the simulations in R. (PDF) [file pone.0078288.s003.pdf]

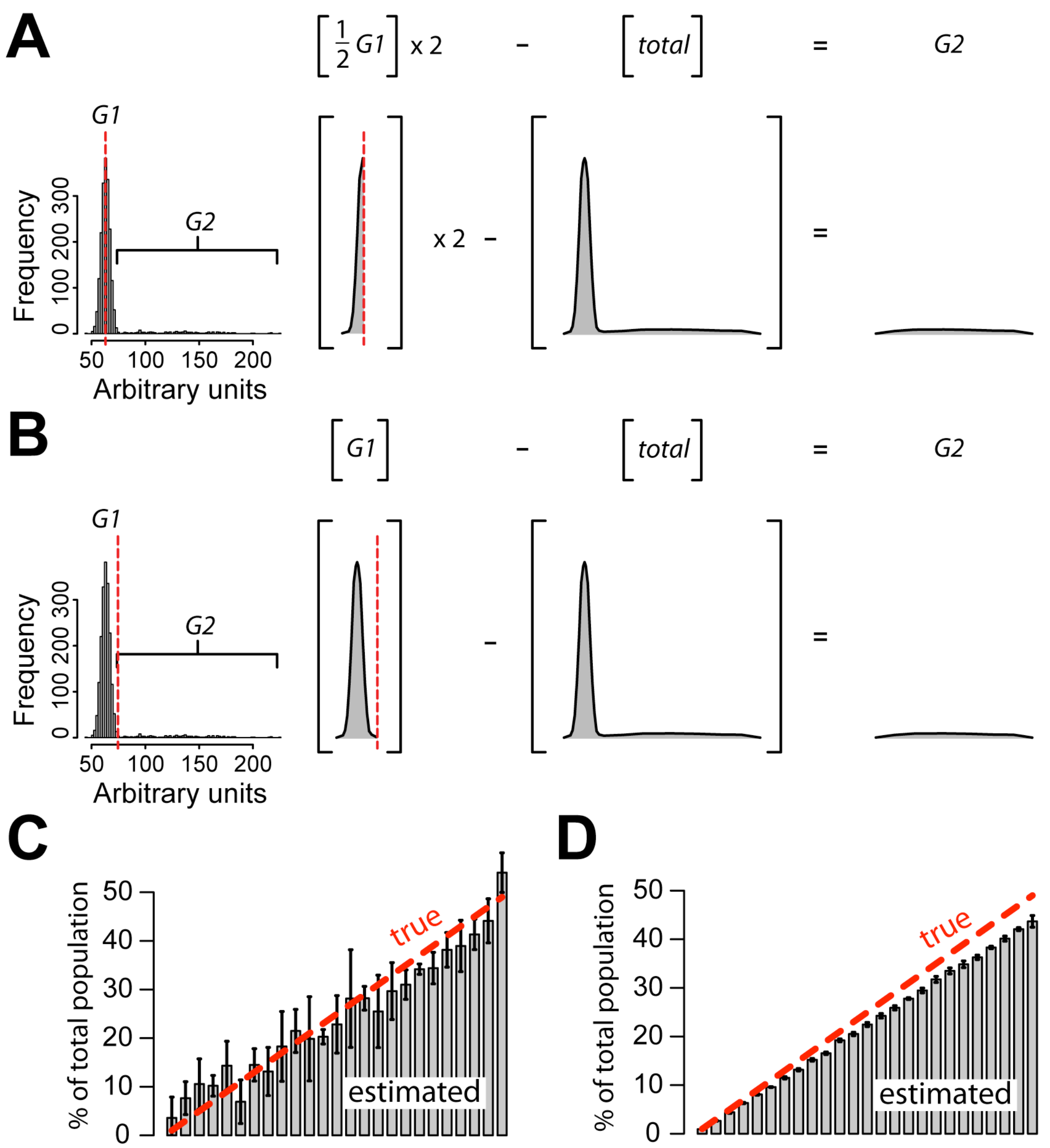

Figure S3. Estimation of the small subpopulation size (percentage of total population) in a bimodal population via visual determination of either the mid-point of the histogram peak (A, C), or alternatively the approximate border between histogram peak and histogram tail (B, D). (A) To calculate the percentage of the small subpopulation the number of observations in *G1* was subtracted from the total number of cells. The *G1* fraction was approximated by quantifying the “left” half of the peak mid-point (dotted line), and multiplying by 2. This approach is similar to the “hand-analysis method” proposed by Bates and colleagues [1] [2]. (B) Same as (A) but *G1* fraction was approximated by quantifying the “left” part of the peak-tail border (dotted line). This approach is similar to the *Manual* method as proposed in this paper (Protocol S1). Left panels in (A) and (B) show a histogram of a simulated bimodal population with standard deviations 3.9 and 37.7, mean values 63 and 127.3, and number of observations 1860 and 140, for *G1* and *G2* Gaussian fractions, respectively. Gaussian simulations were carried out by the *R* function *rnorm(...)*. (C) Simulation results of estimates of a range of true suppopulation sizes (dotted line; 1-49%) using the method according to (A) and implemented in the *R* function *sim.midpoint(...)* (*R* code 1, see below). D) Same as (C) but method according to (B) implemented in the *R* function *sim.peaktail(...)* (*R* code 2, see below). Error bars denote SD (calculated from 5 independent repetitions).

Accuracy of histogram-based manual method of subpopulations determination.

	**Peak mid-point determination[1]**		**Peak-tail border determination[1]**	
**True subpopulation size (%)[2]**	**Estimated subpopulation size (%)[3]**	**Bias[4]**	**Estimated subpopulation size (%)[3]**	**Bias[4]**
1.0	3.6 ± 4.3	260.0 ± 428.4	0.9 ± 0.0	-7.0 ± 4.5
3.0	7.7 ± 3.4	155.3 ± 112.8	2.7 ± 0.1	-11.3 ± 3.2
5.0	10.6 ± 5.2	111.2 ± 103.4	4.4 ± 0.2	-11.6 ± 4.6
7.0	10.2 ± 2.1	45.7 ± 30.4	6.3 ± 0.1	-9.7 ± 1.6
9.0	14.3 ± 5.0	59.3 ± 55.7	8.1 ± 0.2	-10.1 ± 1.7
11.0	6.9 ± 4.5	-37.1 ± 40.7	9.6 ± 0.1	-12.7 ± 0.6
13.0	14.5 ± 3.3	11.5 ± 25.6	11.5 ± 0.2	-11.5 ± 1.8
15.0	13.1 ± 5.0	-12.4 ± 33.0	13.2 ± 0.2	-12.2 ± 1.7
17.0	18.3 ± 7.2	7.7 ± 42.2	15.2 ± 0.3	-10.6 ± 1.6
19.0	21.5 ± 4.4	13.1 ± 23.3	16.6 ± 0.3	-12.9 ± 1.4
21.0	19.9 ± 8.7	-5.4 ± 41.2	19.2 ± 0.3	-8.5 ± 1.3
23.0	20.3 ± 1.5	-11.8 ± 6.5	20.5 ± 0.4	-10.8 ± 1.5
25.0	22.8 ± 5.9	-8.6 ± 23.6	22.5 ± 0.4	-10.1 ± 1.7
27.0	28.1 ± 10.0	4.2 ± 37.2	24.3 ± 0.5	-10.2 ± 1.7
29.0	28.2 ± 2.4	-2.8 ± 8.4	25.9 ± 0.4	-10.7 ± 1.6
31.0	25.5 ± 7.5	-17.7 ± 24.0	27.8 ± 0.2	-10.4 ± 0.7
33.0	29.7 ± 5.8	-10.1 ± 17.7	29.5 ± 0.5	-10.7 ± 1.5
35.0	31.0 ± 3.0	-11.4 ± 8.6	31.8 ± 0.6	-9.3 ± 1.8
37.0	34.2 ± 1.1	-7.6 ± 2.9	33.5 ± 0.6	-9.5 ± 1.7
39.0	34.4 ± 3.2	-11.8 ± 8.3	34.9 ± 0.7	-10.6 ± 1.8
41.0	38.2 ± 3.5	-6.9 ± 8.7	36.3 ± 0.5	-11.5 ± 1.1
43.0	39.0 ± 5.3	-9.4 ± 12.3	38.3 ± 0.2	-10.9 ± 0.6
45.0	41.3 ± 3.1	-8.1 ± 7.0	40.1 ± 0.5	-10.8 ± 1.2
47.0	44.1 ± 4.5	-6.1 ± 9.6	42.1 ± 0.3	-10.5 ± 0.6
49.0	54.0 ± 4.1	10.3 ± 8.3	43.7 ± 1.2	-10.9 ± 2.5

1) Methods of subpopulation detection (see Additional file 22 Figure 1 A and B).

2) True subpopulations were simulated (see Additional file 22 *R* code) using the *R* function *rnorm(...)* with a standard deviation of 37.7, a mean value of 127.3, and the number of observations corresponding to the subpopulation percentage to be tested from a total number of 2000 observations. Mean and standard deviation used were obtained from fluorescence microscopy analysis of batch grown *P. knackmussii* P_{int}-*egfp* in 3CBA (see Additional file 5).

3) Estimated subpopulations (mean ± SD; 5 independent repetitions) also shown in Additional file Figure 1C, D. Were determined by quantifying the larger subpopulation in a bimodal population and subtracting it from the total population. This was either done by peak mid-point determination or by peak-tail border determination (see Additional file 22 Figure 1A,B for details) using the *R* function *sim.midpoint(...)* and *sim.peaktail(...)*, repectively (see Additional file 22 *R* code 1, 2). The bimodal population was simulated by mixing two simulated populations; a true subpopulation[2] and a second subpopulation. The second subpopulation was created using the *R* function *rnorm(...)* with a standard deviation of 3.9, a mean value of 63.0, and the number of observations depending on the sample size of true subpopulation[2] to give a total of 2000 observations. Mean and standard deviation used were obtained from fluorescence microscopy analysis of batch grown *P. knackmussii* P_{int}-*egfp* in 3CBA (see Additional file 5).

4) Bias was calculated according to: (estimated subpopulation size – true subpopulation size)/true subpopulation size x 100

***R* code 1**. *R* code defining the function *sim.midpoint(...)*, which simulates bimodal populations and estimates subpopulation size according to histogram peak mid-point determination (see Additional file 22 Figure 1A).

```
#vector of subpopulation sizes to be tested
my.true.fractions<-rep(seq(1,50,2),each=5)/100

#definition of function
sim.midpoint<- function(total.N = 2000, true.fractions = my.true.fractions) {
		true.size<-true.fractions*total.N
		sim.list<-list()
		for (i in 1:length(true.fractions)) {

				a<-rnorm(total.N-total.N*true.fractions[i],63,3.9)#subpopulation1
				b<-rnorm(total.N*true.fractions[i],127.3,37.7)#subpopulation2
				my.sim<-c(a,b)#mixed bimodal population

				sim.list[[i]] <- my.sim
				}

		my.results<-numeric()
		for (i in 1:length(sim.list)) {
				sim.index<-sim.list[[i]]
				hist(sim.list[[i]],breaks=100, col="grey", main=paste(i,"of",length(sim.list)))
				my.estimate <-locator(n=1)#mouse-click determination of histogram peak mid-point
				dev.off()
				my.results[i]<-length(sim.index)-length(sim.index[sim.index<my.estimate$x])*2
				}
		midpoint.data<<-cbind(my.results, true.size)/20
		midpoint.data
}
sim.midpoint()#execution of function
```

***R* code 2**. *R* code defining the function *sim.peaktail(...)*, which simulates bimodal populations and estimates subpopulation size according to histogram peak-tail border determination (see Additional file 22 Figure 1A).

```
#vector of subpopulation sizes to be tested
my.true.fractions<-rep(seq(1,50,2),each=5)/100
#definition of function
sim.peaktail<- function(total.N = 2000, true.fractions = my.true.fractions ) {
        true.size<-true.fractions*total.N
        sim.list<-list()
        for (i in 1:length(true.fractions)) {

                a<-rnorm(total.N-total.N*true.fractions[i],63,3.9)#subpopulation1
                b<-rnorm(total.N*true.fractions[i],127.3,37.7)#subpopulation2
                my.sim<-c(a,b)#mixed bimodal population

                sim.list[[i]] <- my.sim
                }

        my.results<-numeric()
        for (i in 1:length(sim.list)) {
                sim.index<-sim.list[[i]]
                hist(x=sim.index,breaks=100, col="grey", main=paste(i,"of",length(sim.list)))
                my.estimate <-locator(n=1)#mouse-click determination of histogram peak-tail border
                dev.off()
                my.results[i]<-length(sim.index)-length(sim.index[sim.index<my.estimate$x])
                }
                peaktail.data<<-cbind(my.results, true.size)/20
                peaktail.data
}
sim.peaktail()#execution of function
```

References

1. Bates D, Epstein J, Boye E, Fahrner K, Berg H, Kleckner N: **The *Escherichia coli* baby cell column: a novel cell synchronization method provides new insight into the bacterial cell cycle**. *Mol Microbiol* 2005, **57**(2):380-391.
2. Bates D, Kleckner N: **Chromosome and Replisome Dynamics in *E. coli*: Loss of Sister Cohesion Triggers Global Chromosome Movement and Mediates Chromosome Segregation**. *Cell* 2005, **121**(6):899-911.
