# Supplementary material for: Improved Statistical Analysis of Low Abundance Phenomena in Bimodal Bacterial Populations
Source: PLoS One. 2013 Oct 30;8(10):e78288. doi: 10.1371/journal.pone.0078288 (PMC3813492; doi:10.1371/journal.pone.0078288)
Supplement: Figure S4 — The use of P-P plots for non-parametric and graphical response quantification. This file illustrates the concept of employing P-P plots for non-parametric and graphical response quantification [46], using results obtained from the measurement of ICEclc activity in P. knackmussii B13 grown under different environmental conditions as an example data-set. (PDF) [file pone.0078288.s004.pdf]

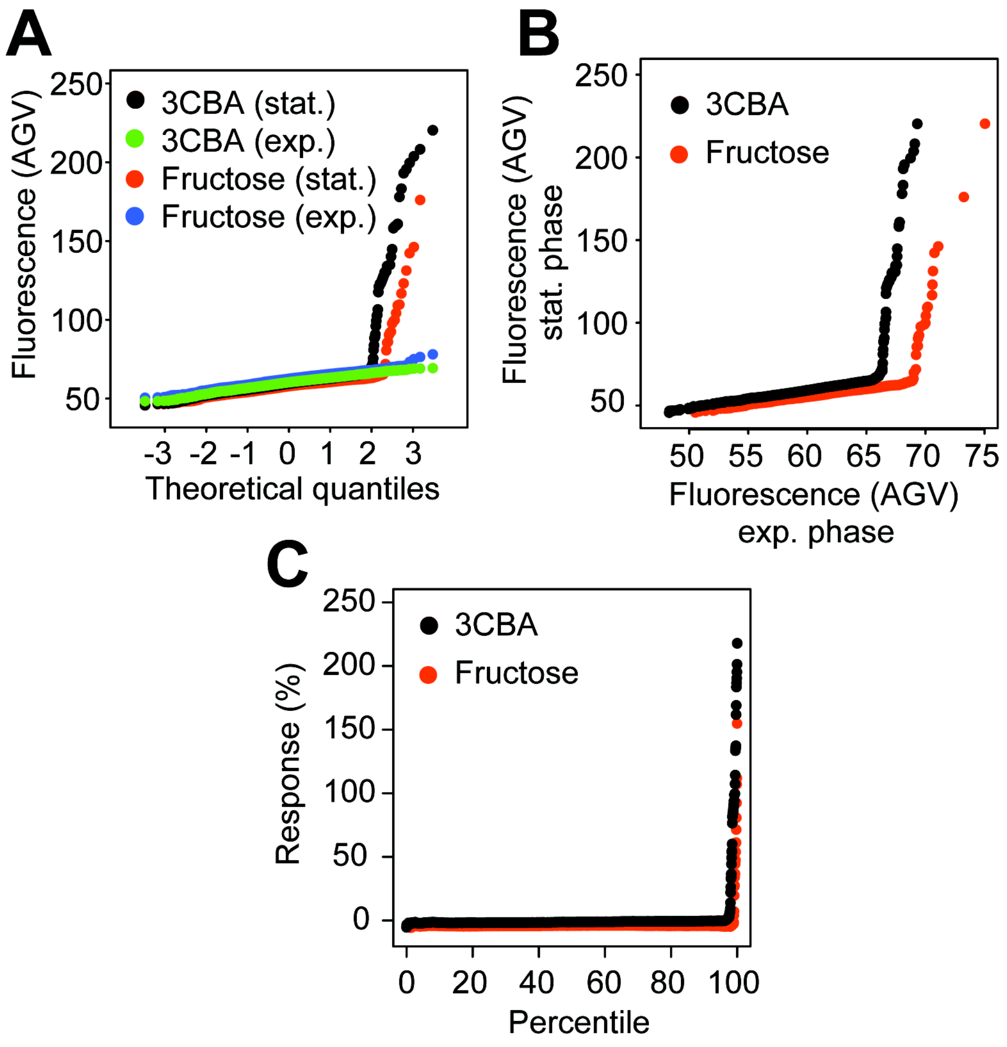

Figure S4. (A) P-P plot representation of single cell eGFP fluorescence from P_{int}-*egfp* in *P. knackmussii* B13 corresponding to ICE*clc* activation in response to different growth conditions. Black, stationary (stat.) phase after growth on 3CBA (5 mM); red, stat. phase after growth on fructose (5 mM); green, exponential (exp.) phase with growth on 3CBA (5 mM); blue, exp. phase with growth on fructose (5 mM). (B) P-P plot representation of data shown in (A) plotting ranked values of stat. phase fluorescence against ranked values of exp. phase fluorescence. (C) ICE*clc* activity-response in *Pseudomonas knackmussi* B13 to pre-growth on fructose or 3CBA, quantified over the full percentile range (also see Additional file 23 Table 1). A similar quantification method was proposed by McArthur and collegues [1]. For each carbon source, response was calculated from the data sets shown in (A), and according to $100 \times \left[\left(P_k^{STAT} - P_k^{EXP}\right) / P_k^{EXP}\right]$, where P_k^{STAT} is the *k*th percentile at stat. phase and P_k^{Exp} is the *k*th percentile at exp. phase. Thus, the absence of any subpopulation activity in the exp. populations provide a negative control population, by which subpopulation activity in the stat. fractions may be assessed. Black, response to pre-growth on 3CBA (5mM). Red, response to pre-growth on fructose (5mM). In this figure, a positive response corresponds to cell fluorescence stonger in stationary phase than in exponential phase, while a negative response corresponds to vice versa.

References

1. MacArthur B, D., Tare R, S., Please CP, Prescott P, Oreffo R, O., C.: **A non-invasive method for *in situ* quantification of subpopulation behaviour in mixed cell culture**. *J R Soc Interface* 2006, **3**(6):63-69.
