# Supplementary material for: Improved Statistical Analysis of Low Abundance Phenomena in Bimodal Bacterial Populations
Source: PLoS One. 2013 Oct 30;8(10):e78288. doi: 10.1371/journal.pone.0078288 (PMC3813492; doi:10.1371/journal.pone.0078288)
Supplement: Table S1 — Bacterial strains used in this work. In this file we provide a list with all bacterial strains used in this work. (DOC) [file pone.0078288.s005.doc]

**Table S1.** Strains used in this study.

| **Strain** | **Description** | **Reference** |
| --- | --- | --- |
| *P. knackmussii* B13-1343 | P_int_*-egfp* | [1] |
| *P. knackmussii* B13-2396 | P_inR_*-egfp* | [2] |
| *P. knackmussii* B13-2398 |
| *P. knackmussii* B13-2399 |
| *P. putida* UWC-3408 | ICE*clc*, P_int_*-egfp* | [3] |

**References**

1. Sentchilo V, Ravatn R, Werlen C, Zehnder AJ, van der Meer JR: **Unusual integrase gene expression on the *clc* genomic island in *Pseudomonas* sp. Strain B13**. *Journal of Bacteriology* 2003, **185**(15):4530-4538.

2. Minoia M, Gaillard M, Reinhard F, Stojanov M, Sentchilo V, van der Meer JR: **Stochasticity and bistability in horizontal transfer control of a genomic island in *Pseudomonas***. *Proc Natl Acad Sci U S A* 2008, **105**(52):20792-20797.

3. Reinhard F, Miyazaki R, Pradervand N, van der Meer JR: **Cell differentiation to "mating bodies" induced by an integrating and conjugative element in free-living bacteria**. *Curr Biol* 2013, **23**(3):255-259.
