# Supplementary material for: Improved Statistical Analysis of Low Abundance Phenomena in Bimodal Bacterial Populations
Source: PLoS One. 2013 Oct 30;8(10):e78288. doi: 10.1371/journal.pone.0078288 (PMC3813492; doi:10.1371/journal.pone.0078288)
Supplement: Table S2 — Pseudomonas knackmussii B13 growth in batch culture. This file contains a table listing timing of exponential growth and onset of stationary phase in batch cultures of P. knackmussii B13 and P. putida UWC (ICEclc) grown on different carbon substrates. (DOC) [file pone.0078288.s006.doc]

**Table S2.** *Pseudomonas knackmussi* B13 growth as determined via culture turbidity at 600 nm.

| **Strain** | **Carbon substrate** | **Exponential growth (h)^1^** | **Onset of stationary phase (h)^1^** |
| --- | --- | --- | --- |
| *P. knackmussi* B13-1343 | 3-chlorobenzoate (5 mM) | 8 - 20 | 24 |
|  | glucose (10 mM) | 2 - 8 | 12 |
|  | fructose (10 mM) | 20 - 40 | 45 |
|  | benzoate (10 mM) | 4 - 10 | 12 |
|  | 4-hydroxybenzoate (10 mM) | 1 - 15 | 18 |
|  | anthranilate (5 mM) | 40 - 48 | 50 |
| *P. putida* UWC (ICE*clc*) - 2508 | 3-chlorobenzoate (5 mM) | 30 - 50 | 60 |

1) Time after inocculation.
