# Supplementary material for: Improved Statistical Analysis of Low Abundance Phenomena in Bimodal Bacterial Populations
Source: PLoS One. 2013 Oct 30;8(10):e78288. doi: 10.1371/journal.pone.0078288 (PMC3813492; doi:10.1371/journal.pone.0078288)
Supplement: Table S3 — Large and small subpopulation parameters of fluorescence data from promoter- egfp reporters for ICE clc activation in Pseudomonas knackmussii B13. This file contains a data table showing typical measured large and small subpopulation parameters of fluorescence data obtained from promoter-egfp reporters for ICEclc activation in P. knackmussii B13 after growth on 3CBA. These parameters were used as reference parameters for ICEclc activation when creating some of the simulated subpopulations in Figures 7, 8, 9 and 10, Table 2, and Figure S2, S3. (DOC) [file pone.0078288.s007.doc]

**Table S3.** Reference subpopulation parameters calculated from single cell fluorescence values obtained from P_inR_*-egfp* reporter in *P. knackmussii* B13.

|  | **Large subpopulation^1^** | | **Small subpopulation^1^** | |  |  |
| --- | --- | --- | --- | --- | --- | --- |
| **Strain (promoter-*egfp* reporter)** | **SD^2^** | **Mean^2^** | **SD^2^** | **Mean^2^** | **Mean difference (ratio)^2,3^** | **Proportion (%)^4^** |
| *P. knackmussii* B13-1343  (P_int_*-egfp*) | 4.7 | 70.0 | 27.1 | 109.5 | 39.5 (1.6) | 4.2 |
| *P. knackmussii* B13-2396  (P_inR_*-egfp*) | 3.2 | 60.3 | 44.2 | 142.2 | 81.9 (2.4) | 3.0 |
| *P. knackmussii* B13-2397  (P_inR_*-egfp*) | 3.0 | 59.4 | 34.1 | 125.1 | 65.7 (2.1) | 2.0 |
| *P. knackmussii* B13-2398  (P_inR_*-egfp*) | 5.2 | 66.1 | 38.8 | 121.5 | 55.4 (1.8) | 4.0 |
| *P. knackmussii* B13-2399  (P_inR_*-egfp*) | 3.3 | 59.3 | 44.3 | 138.3 | 79.0 (2.3) | 3.3 |
| **Mean population parameter^5^** | **3.9** | **63.0** | **37.7** | **127.3** | **64.3 (2.0)** | **3.3** |

1) Large and small subpopulation were separated according to the *Boxplot3* method. Fluorescence values of at least 2000 cells in stationary phase were investigated per strain after 25 hours of batch growth on 3CBA (5mM).

2) eGFP fluorescence (arbitrary units)

3) Difference between the means of large and small subpopulation (arbitrary units).

4) Proportion of small subpopulation as determined by the *Boxplot1.5* method.

5) Mean estimated population parameters as calculated from the four P_inR_*-egfp* strains only. These were used as reference parameters for the creation of the bimodal simulations in this paper.
