# Supplementary material for: Improved Statistical Analysis of Low Abundance Phenomena in Bimodal Bacterial Populations
Source: PLoS One. 2013 Oct 30;8(10):e78288. doi: 10.1371/journal.pone.0078288 (PMC3813492; doi:10.1371/journal.pone.0078288)
Supplement: Table S4 — Significance testing of subpopulation effects from ICE clc activation under different conditions quantified by different PS methods. This file contains a data table showing results from quantifications of small subpopulation effects by different PS methods. Results from this table are visualized in Figure 3A. (DOC) [file pone.0078288.s008.doc]

**Table S4.** Proportions of subpopulations as calculated via four different PS methods from single cell eGFP fluorescence data.

|  |  |  | **Subpopulation size (%)^3^** | | | |  |  |  |
| --- | --- | --- | --- | --- | --- | --- | --- | --- | --- |
| **Category^1^** | **Carbon source** | **Method^2^** | **P_inR_*-egfp* 2396^4^** | **P_inR_*-egfp* 2398^4^** | **P_inR_*-egfp* 2399^4^** | **P_int_*-egfp* 1343^4^** | **Mean**  **(± SD)** | **Welch Two Sample t-test^5^** | **Wilcoxon rank sum test^5^** |
| A | 3CBA | *Default* | 2.7 | 5.1 | 2.8 | 4.7 | 3.8 ± 1.3 | B*, C* | B*, C* |
|  |  | *Manual* | 2.8 | 3.4 | 3.3 | 4.6 | 3.5 ± 0.8 | B**, C** | B*, C* |
|  |  | *Boxplot1.5* | 2.8 | 4.3 | 2.8 | 4.3 | 3.6 ± 0.9 | B*, C** | B*, C** |
|  |  | *Boxplot3* | 2.6 | 2.7 | 2.5 | 3.2 | 2.8 ± 0.3 | B**, C** | B*, C* |
|  |  |  |  |  |  |  |  |  |  |
| B | Fructose | *Default* | 1.3 | 0.9 | 1.2 | 2.4 | 1.5 ± 0.7 | A* | A* |
|  |  | *Manual* | 1.2 | 0.9 | 1.2 | 2.1 | 1.4 ± 0.5 | A**, C* | A*, C* |
|  |  | *Boxplot1.5* | 1.2 | 0.9 | 1.2 | 2.4 | 1.4 ± 0.7 | A* | A* |
|  |  | *Boxplot3* | 1.1 | 0.9 | 1.1 | 1.8 | 1.2 ± 0.4 | A**, C* | A**, C* |
|  |  |  |  |  |  |  |  |  |  |
| C | Glucose | *Default* | 0.6 | 0.6 | 0.9 | 2.0 | 1.0 ± 0.7 | A* | A* |
|  |  | *Manual* | 0.4 | 0.1 | 0.3 | 0.6 | 0.4 ± 0.2 | A**, B* | A*, B* |
|  |  | *Boxplot1.5* | 0.5 | 0.5 | 0.8 | 1.6 | 0.9 ± 0.5 | A** | A** |
|  |  | *Boxplot3* | 0.4 | 0.1 | 0.3 | 0.6 | 0.4 ± 0.2 | A**, B* | A*, B* |

Data was obtained from P_int_*-egfp* or P_inR_-*egfp* expression in *P. knackmussii* B13 batch-grown to stationary phase with either 3CBA, fructose or glucose as carbon source (also see Figure 4 A).

1) Categories for significance testing

2) PS method used to determine subpopulation.

3) Subpopulation size (percent of total population)

4) Reporter-*egfp* construct and *P. knackmussii* B13 strain number.

5) Significance testing comparing the means between different categories of the same methods. * and ** indicate significant differences at P<0.05 and P<0.01, respectively.
