# Supplementary material for: Improved Statistical Analysis of Low Abundance Phenomena in Bimodal Bacterial Populations
Source: PLoS One. 2013 Oct 30;8(10):e78288. doi: 10.1371/journal.pone.0078288 (PMC3813492; doi:10.1371/journal.pone.0078288)
Supplement: Table S5 — Significance testing of subpopulation effects from ICE clc activation under different conditions quantified by different non-PS methods. This file contains a data table showing results from quantifications of small subpopulation effects by different PS methods. Results from this table are visualized in Figure 3B. (DOC) [file pone.0078288.s009.doc]

**Table S5** Proportions of subpopulations as calculated via four different non-PS methods from single cell eGFP fluorescence data.

|  |  |  | **eGFP fluorescence** | | | |  |  |  |
| --- | --- | --- | --- | --- | --- | --- | --- | --- | --- |
| **Category^1^** | **Carbon source** | **Method^2^** | **P_inR_*-egfp* 2396^3^** | **P_inR_*-egfp* 2398^3^** | **P_inR_*-egfp* 2399^3^** | **P_int_*-egfp* 1343^3^** | **Mean**  **(± SD)** | **Welch Two Sample t-test^4^** | **Wilcoxon rank sum test^4^** |
| A | 3CBA | *Mean* | 62.5 | 68.8 | 61.4 | 72.1 | 66.2 ± 5.1 |  |  |
|  |  | *95 Percentile* | 66.2 | 79.7 | 65.0 | 81.7 | 73.1 ± 8.7 |  |  |
|  |  | *Mean top 5%* | 107.5 | 120.6 | 106.5 | 111.6 | 111.6 ± 6.4 | B**, C** | B*, C* |
|  |  | *Boosted Mean* | 64.1 | 73.4 | 63.0 | 76.2 | 69.2 ± 6.6 |  |  |
|  |  |  |  |  |  |  |  |  |  |
| B | Fructose | *Mean* | 60.7 | 58.4 | 58.1 | 64.4 | 66.2 ± 5.1 |  |  |
|  |  | *95 Percentile* | 64.5 | 62.2 | 62.2 | 70.9 | 65.0 ± 4.1 |  |  |
|  |  | *Mean top 5%* | 76.6 | 72.0 | 75.3 | 84.6 | 77.1 ± 5.4 | A** | A* |
|  |  | *Boosted Mean* | 63.2 | 61.0 | 60.7 | 67.9 | 63.2 ± 3.3 |  |  |
|  |  |  |  |  |  |  |  |  |  |
| C | Glucose | *Mean* | 62.2 | 59.7 | 61.0 | 62.8 | 61.4 ± 1.4 |  |  |
|  |  | *95 Percentile* | 68.0 | 66.1 | 67.1 | 69.6 | 67.7 ± 1.5 |  |  |
|  |  | *Mean top 5%* | 74.5 | 69.3 | 72.2 | 76.5 | 73.1 ± 3.1 | A** | A* |
|  |  | *Boosted Mean* | 66.2 | 63.7 | 64.8 | 66.8 | 65.4 ± 1.4 |  |  |

Data was obtained from P_int_*-egfp* or P_inR_-*egfp* expression in *P. knackmussii* B13 batch-grown to stationary phase with either 3CBA, fructose or glucose as carbon source (also see Figure 4 B).

1) Categories for significance testing

2) PS method used to determine subpopulation.

3) Reporter-*egfp* construct and *P. knackmussii* B13 strain number.

4) Significance testing comparing the means between different categories of the same methods. * and ** indicate significant differences at P<0.05 and P<0.01, respectively.
