# Supplementary material for: Improved Statistical Analysis of Low Abundance Phenomena in Bimodal Bacterial Populations
Source: PLoS One. 2013 Oct 30;8(10):e78288. doi: 10.1371/journal.pone.0078288 (PMC3813492; doi:10.1371/journal.pone.0078288)
Supplement: Table S6 — Accuracy as a function of subpopulation proportion (range: 0.1–40%; n = 40) at a mean difference of 67.8 and a subpopulation standard deviation of 37.7. This file contains a data table showing numerical data corresponding to Figure 8. (DOC) [file pone.0078288.s010.doc]

**Table S6.** Simulation results corresponding to Figure 8 with *Boxplot1.5* or *Boxplot 3.0* as method of subpopulation determination.

| **Boxplot 1.5** | | | | | | **Boxplot 3.0** | | | | | |
| --- | --- | --- | --- | --- | --- | --- | --- | --- | --- | --- | --- |
| **Figure 8A** |  | **Figure 8B** |  | **Figure 8C** |  | **Figure 8A** |  | **Figure 8B** |  | **Figure 8C** |  |
| **Proportion** | **Accuracy^1^** | **Mean.diff** | **Accuracy^1^** | **SD** | **Accuracy^1^** | **Proportion** | **Accuracy^1^** | **Mean.diff** | **Accuracy^1^** | **SD** | **Accuracy^1^** |
| 0.10 | 200.00 | 2.00 | -58.73 | 10.00 | 3.17 | 0.10 | 0.00 | 2.00 | -68.25 | 10.00 | 0.00 |
| 1.10 | 13.64 | 5.46 | -57.14 | 11.03 | 3.17 | 1.10 | 0.00 | 5.46 | -55.56 | 11.03 | 0.00 |
| 2.10 | 4.76 | 8.92 | -46.03 | 12.05 | 3.17 | 2.10 | -7.14 | 8.92 | -55.56 | 12.05 | 0.00 |
| 3.15 | -6.35 | 12.38 | -42.86 | 13.08 | 3.17 | 3.15 | -17.46 | 12.38 | -61.90 | 13.08 | 0.00 |
| 4.15 | -3.61 | 15.85 | -41.27 | 14.10 | 3.17 | 4.15 | -6.02 | 15.85 | -63.49 | 14.10 | 0.00 |
| 5.20 | -6.73 | 19.31 | -41.27 | 15.13 | 3.17 | 5.20 | -7.69 | 19.31 | -42.86 | 15.13 | 0.00 |
| 6.20 | -5.65 | 22.77 | -25.40 | 16.15 | 3.17 | 6.20 | -8.06 | 22.77 | -52.38 | 16.15 | 0.00 |
| 7.25 | -7.59 | 26.23 | -23.81 | 17.18 | 3.17 | 7.25 | -11.03 | 26.23 | -36.51 | 17.18 | 0.00 |
| 8.25 | -7.88 | 29.69 | -26.98 | 18.21 | 3.17 | 8.25 | -11.52 | 29.69 | -44.44 | 18.21 | 0.00 |
| 9.30 | -9.14 | 33.15 | -23.81 | 19.23 | 3.17 | 9.30 | -11.29 | 33.15 | -28.57 | 19.23 | 0.00 |
| 10.30 | -8.25 | 36.62 | -22.22 | 20.26 | 3.17 | 10.30 | -7.77 | 36.62 | -31.75 | 20.26 | 0.00 |
| 11.35 | -6.17 | 40.08 | -19.05 | 21.28 | 3.17 | 11.35 | -13.66 | 40.08 | -26.98 | 21.28 | -1.59 |
| 12.35 | -7.29 | 43.54 | -15.87 | 22.31 | 3.17 | 12.35 | -11.74 | 43.54 | -30.16 | 22.31 | -1.59 |
| 13.35 | -6.74 | 47.00 | -9.52 | 23.33 | 1.59 | 13.35 | -13.11 | 47.00 | -22.22 | 23.33 | 0.00 |
| 14.40 | -8.68 | 50.46 | -6.35 | 24.36 | 3.17 | 14.40 | -13.89 | 50.46 | -19.05 | 24.36 | -1.59 |
| 15.40 | -8.12 | 53.92 | -7.94 | 25.38 | 1.59 | 15.40 | -10.39 | 53.92 | -14.29 | 25.38 | -1.59 |
| 16.45 | -8.81 | 57.38 | -1.59 | 26.41 | 1.59 | 16.45 | -10.94 | 57.38 | -19.05 | 26.41 | -4.76 |
| 17.45 | -7.16 | 60.85 | -4.76 | 27.44 | 0.00 | 17.45 | -10.60 | 60.85 | -14.29 | 27.44 | -6.35 |
| 18.50 | -8.38 | 64.31 | -3.17 | 28.46 | 1.59 | 18.50 | -13.24 | 64.31 | -12.70 | 28.46 | -4.76 |
| 19.50 | -10.51 | 67.77 | -6.35 | 29.49 | 1.59 | 19.50 | -13.85 | 67.77 | -17.46 | 29.49 | -4.76 |
| 20.55 | -6.33 | 71.23 | -3.17 | 30.51 | 0.00 | 20.55 | -13.63 | 71.23 | -9.52 | 30.51 | -7.94 |
| 21.55 | -9.98 | 74.69 | 1.59 | 31.54 | 0.00 | 21.55 | -15.08 | 74.69 | -9.52 | 31.54 | 0.00 |
| 22.60 | -8.41 | 78.15 | 3.17 | 32.56 | 1.59 | 22.60 | -15.93 | 78.15 | -6.35 | 32.56 | -6.35 |
| 23.60 | -10.17 | 81.62 | 1.59 | 33.59 | -4.76 | 23.60 | -21.19 | 81.62 | -4.76 | 33.59 | -6.35 |
| 24.65 | -9.13 | 85.08 | 3.17 | 34.62 | -7.94 | 24.65 | -19.07 | 85.08 | -3.17 | 34.62 | -6.35 |
| 25.65 | -14.42 | 88.54 | 1.59 | 35.64 | 1.59 | 25.65 | -19.49 | 88.54 | -3.17 | 35.64 | -14.29 |
| 26.70 | -17.79 | 92.00 | 1.59 | 36.67 | -1.59 | 26.70 | -32.02 | 92.00 | -1.59 | 36.67 | -12.70 |
| 27.70 | -40.79 | 95.46 | 1.59 | 37.69 | -6.35 | 27.70 | -50.00 | 95.46 | -6.35 | 37.69 | -17.46 |
| 28.70 | -43.73 | 98.92 | 3.17 | 38.72 | -1.59 | 28.70 | -90.77 | 98.92 | 0.00 | 38.72 | -11.11 |
| 29.75 | -65.21 | 102.38 | 1.59 | 39.74 | -3.17 | 29.75 | -86.72 | 102.38 | 0.00 | 39.74 | -11.11 |
| 30.75 | -64.88 | 105.85 | 3.17 | 40.77 | -1.59 | 30.75 | -97.72 | 105.85 | -1.59 | 40.77 | -7.94 |
| 31.80 | -78.14 | 109.31 | 3.17 | 41.79 | -7.94 | 31.80 | -97.48 | 109.31 | 0.00 | 41.79 | -15.87 |
| 32.80 | -81.71 | 112.77 | 3.17 | 42.82 | -3.17 | 32.80 | -98.63 | 112.77 | -1.59 | 42.82 | -7.94 |
| 33.85 | -88.77 | 116.23 | 3.17 | 43.85 | -6.35 | 33.85 | -99.70 | 116.23 | -3.17 | 43.85 | -15.87 |
| 34.85 | -90.82 | 119.69 | 3.17 | 44.87 | -3.17 | 34.85 | NA | 119.69 | 0.00 | 44.87 | -19.05 |
| 35.90 | -91.50 | 123.15 | 3.17 | 45.90 | -11.11 | 35.90 | NA | 123.15 | 0.00 | 45.90 | -12.70 |
| 36.90 | -95.93 | 126.62 | 3.17 | 46.92 | -6.35 | 36.90 | -99.86 | 126.62 | 0.00 | 46.92 | -9.52 |
| 37.95 | -97.63 | 130.08 | 3.17 | 47.95 | -11.11 | 37.95 | NA | 130.08 | 0.00 | 47.95 | -15.87 |
| 38.95 | -96.66 | 133.54 | 3.17 | 48.97 | -3.17 | 38.95 | NA | 133.54 | 0.00 | 48.97 | -15.87 |
| 40.00 | -96.75 | 137.00 | 3.17 | 50.00 | -3.17 | 40.00 | NA | 137.00 | 0.00 | 50.00 | -22.22 |

1) Accuracy as the percent difference between the estimated and true value.
