# Supplementary material for: Improved Statistical Analysis of Low Abundance Phenomena in Bimodal Bacterial Populations
Source: PLoS One. 2013 Oct 30;8(10):e78288. doi: 10.1371/journal.pone.0078288 (PMC3813492; doi:10.1371/journal.pone.0078288)
Supplement: Table S7 — Accuracy as function of subpopulation proportion (range: 0.1–1.2%; n = 15) at a mean difference of 67.8 and a subpopulation standard deviation of 37.7. Data table corresponding to Figure 10. (DOC) [file pone.0078288.s011.doc]

**Table S7.** Simulation results corresponding to Figure 10.

|  | **Figure 10A (n=2000)** | | **Figure 10B (n=20000)** | | **Figure 10C (n=200000)** | |
| --- | --- | --- | --- | --- | --- | --- |
| **method** | **Proportion** | **Accuracy^1^** | **Proportion** | **Accuracy** | **Proportion** | **Accuracy** |
| *Boxplot1.5* | 0.10 | 100.00 | 0.10 | 390.00 | 0.10 | 351.50 |
|  | 0.15 | 66.67 | 0.18 | 225.71 | 0.18 | 189.92 |
|  | 0.25 | 20.00 | 0.26 | 145.10 | 0.26 | 130.16 |
|  | 0.30 | 16.67 | 0.34 | 113.43 | 0.34 | 96.72 |
|  | 0.40 | 12.50 | 0.41 | 93.90 | 0.41 | 76.81 |
|  | 0.45 | 11.11 | 0.49 | 65.31 | 0.49 | 63.25 |
|  | 0.55 | 9.09 | 0.57 | 60.53 | 0.57 | 52.71 |
|  | 0.60 | 8.33 | 0.64 | 52.71 | 0.65 | 43.80 |
|  | 0.70 | -7.14 | 0.73 | 45.52 | 0.73 | 38.16 |
|  | 0.80 | 0.00 | 0.80 | 40.99 | 0.81 | 34.57 |
|  | 0.85 | 5.88 | 0.88 | 37.29 | 0.89 | 30.55 |
|  | 0.95 | 0.00 | 0.96 | 32.81 | 0.96 | 25.57 |
|  | 1.00 | 0.00 | 1.04 | 32.69 | 1.04 | 24.03 |
|  | 1.10 | 0.00 | 1.12 | 24.55 | 1.12 | 21.10 |
|  | 1.20 | -8.33 | 1.20 | 27.08 | 1.20 | 17.92 |
| *Boxplot3* | 0.10 | 0.00 | 0.10 | -10.00 | 0.10 | -8.50 |
|  | 0.15 | 0.00 | 0.18 | -11.43 | 0.18 | -9.80 |
|  | 0.25 | 0.00 | 0.26 | -5.88 | 0.26 | -9.34 |
|  | 0.30 | 0.00 | 0.34 | -8.96 | 0.34 | -9.84 |
|  | 0.40 | -12.50 | 0.41 | -6.10 | 0.41 | 10.39 |
|  | 0.45 | 0.00 | 0.49 | -9.18 | 0.49 | -9.64 |
|  | 0.55 | -9.09 | 0.57 | -9.65 | 0.57 | -9.54 |
|  | 0.60 | 0.00 | 0.64 | -12.40 | 0.65 | -9.93 |
|  | 0.70 | -7.14 | 0.73 | -11.03 | 0.73 | -9.88 |
|  | 0.80 | -6.25 | 0.80 | -8.70 | 0.81 | -9.54 |
|  | 0.85 | -5.88 | 0.88 | -6.78 | 0.89 | -8.36 |
|  | 0.95 | -21.05 | 0.96 | -10.42 | 0.96 | -9.18 |
|  | 10.00 | -10.00 | 1.04 | -8.17 | 1.04 | -9.78 |
|  | 1.10 | -4.55 | 1.12 | -8.48 | 1.12 | -9.10 |
|  | 1.20 | -8.33 | 1.20 | -7.08 | 1.20 | -9.62 |

1) Accuracy as the percent difference between the estimated and true value.
