# Supplementary material for: Improved Statistical Analysis of Low Abundance Phenomena in Bimodal Bacterial Populations
Source: PLoS One. 2013 Oct 30;8(10):e78288. doi: 10.1371/journal.pone.0078288 (PMC3813492; doi:10.1371/journal.pone.0078288)
Supplement: Table S8 — ICE clc activity-response in Pseudomonas knackmussi B13 Pint- egfp to pre-growth on different carbon sources, quantified over percentile range. Data correspond to Figure S4C. (DOC) [file pone.0078288.s012.doc]

**Table S8.** ICE*clc* activity-response in *Pseudomonas knackmussi* B13 P_int_-*egfp* to pre-growth on different carbon sources, quantified over percentile range. Data correspond to Figure S4C.

|  | **Response (%)^2^** | |
| --- | --- | --- |
| **Percentile^1^** | **Fructose** | **3CBA** |
| 97.80% | -4.74 | 6.47 |
| 97.85% | -4.83 | 6.89 |
| 97.90% | -4.70 | 9.29 |
| 97.95% | -4.75 | 13.97 |
| **98.00%** | -4.59 | **22.02** |
| 98.05% | -4.51 | **26.27** |
| 98.10% | -4.35 | **32.97** |
| 98.15% | -4.19 | **33.72** |
| 98.20% | -3.97 | **36.64** |
| 98.25% | -3.97 | **44.32** |
| 98.30% | -3.98 | **49.31** |
| 98.35% | -4.05 | **53.88** |
| 98.40% | -3.91 | **54.74** |
| 98.45% | -3.73 | **60.10** |
| 98.50% | -3.63 | **76.48** |
| 98.55% | -3.20 | **81.58** |
| 98.60% | -3.17 | **82.25** |
| 98.65% | -3.05 | **84.14** |
| 98.70% | -3.02 | **84.27** |
| 98.75% | -2.73 | **85.43** |
| 98.80% | -2.88 | **86.73** |
| 98.85% | -2.57 | **88.33** |
| 98.90% | -2.59 | **89.67** |
| 98.95% | -1.32 | **91.11** |
| 99.00% | 4.07 | **93.83** |
| 99.05% | 7.13 | **94.03** |
| 99.10% | 19.60 | **94.01** |
| **99.15%** | **26.68** | **98.82** |
| 99.20% | **27.52** | **99.00** |
| 99.25% | **34.04** | **99.61** |
| 99.30% | **35.90** | **99.68** |
| 99.35% | **36.79** | **107.35** |
| 99.40% | **44.43** | **114.22** |
| 99.45% | **46.14** | **133.47** |
| 99.50% | **47.41** | **135.01** |
| 99.55% | **53.87** | **135.87** |
| 99.60% | **60.95** | **137.32** |
| 99.65% | **61.26** | **161.80** |
| 99.70% | **71.40** | **169.08** |
| 99.75% | **80.76** | **183.48** |
| 99.80% | **92.43** | **186.68** |
| 99.85% | **107.00** | **190.35** |
| 99.90% | **111.90** | **195.29** |
| 99.95% | **154.82** | **201.38** |
| 100.00% | **217.95** | **217.84** |
|  |  |  |

1) Percentile range from 97.80-100%. Bold, first percentile value corresponding to response > 20%.

2) Positive values denote cell fluorescence brighter in stationary phase than in exponential phase, while a negative response corresponds to vice versa. Bold, response > 20%.

**References**

1. MacArthur B, D., Tare R, S., Please CP, Prescott P, Oreffo R, O., C.: **A non-invasive method for *in situ* quantification of subpopulation behaviour in mixed cell culture**. *J R Soc Interface* 2006, **3**(6):63-69.
