# Supplementary material for: Improved Statistical Analysis of Low Abundance Phenomena in Bimodal Bacterial Populations
Source: PLoS One. 2013 Oct 30;8(10):e78288. doi: 10.1371/journal.pone.0078288 (PMC3813492; doi:10.1371/journal.pone.0078288)
Supplement: Protocol S1 — Description of R functions for quantification of low abundance phenomena in bimodal populations. This file provides a detailed description of the proposed R functions findsub(…) and get.ci(…) as tools for quantification of small subpopulation phenomena and method confidence interval calculation, respectively. We also show examples of graphical and command-line output from these functions. (PDF) [file pone.0078288.s013.pdf]

Protocol S1

Description of the *R* functions *findsub(...), get.ci(...),* and *get.ci.other(...)*

Different strategies of small subpopulation quantification and robustness testing were translated into the *R* [1] functions *findsub(...)* and *get.ci(...)*, respectively (for code see Protocol S2). In this way subpopulations could be computationally calculated following a simple protocol of commands (Protocol S1, Figure 1: *(5)-(8)*). The functions provide numerical as well as graphical output (Protocol S1, Figure 2-8). Functions used in *Default* or *Manual* mode were designed to work on basis of interactive graphs requiring mouse-clicking at the approximate locations of the large subpopulation minimum and maximum, or at the point of separation between large and small subpopulation, respectively (Protocol S1, Figure 2, 3). In contrast, functions in *Boxplot1.5* and *Boxplot3* modes were designed to run fully automatically once initiated (Protocol S1, Figure 4, 5). An additional mode *Other* was designed to employ other methods of characterization corresponding to the mean, 95th percentile, boosted mean, and the mean top five % of the total population, respectively (Protocol S1, Figure 6). Bootstrap confidence interval calculations may be performed by the functions *get.ci(...)* (Protocol S1, Figure 7, 8) and *get.ci.other(...)*. While the former calculates the confidence intervals of the methods in modes *Default*, *Manual*, *Boxplot1.5*, and *Boxplot3*, the latter calculates confidence intervals of *Other* modes. *get.ci(...)* and *get.ci.other(...)* both employ the *R* funtions *boot(...)* and *boot.ci(...)* included in the *R* package *boot* [2] [3], which perform re-sampling of original data set (with replacement) and subsequent method application. Shown below, are examples of *R* output of the functions *findsub(...)* and *get.ci(...)* as tested on an example data set (Protocol S2). The example data set was compiled from single cell eGFP fluorescence values of *Pseudomonas knackmussi* B13 strain 1343 grown in batch culture on minimal medium with 3-chlorobenzaote as carbon source (10 mM), sampled 10 h after reaching stationary phase.

```
(1)   rm(list=ls(all=TRUE))
(2)   setwd("/Users/My_Folder_Address")

(3)   example.data <- read.table("example.data.txt", header=TRUE)
(4)   source("FINDSUB_code.txt")

(5)   findsub(x=example.data$value, mode="default")
(6)   findsub(x=example.data$value, mode="manual")
(7)   findsub(x=example.data$value, mode="boxplot1.5")
(8)   findsub(x=example.data$value, mode="boxplot3")
(9)   findsub(x=example.data$value, mode="other")

(10)  get.ci(x=example.data$value, trials=20, mode="default")
(11)  get.ci(x=example.data$value, trials=20, mode="manual")
(12)  get.ci(x=example.data$value, R=500, mode="boxplot1.5")
(13)  get.ci(x=example.data$value, R=500, mode="boxplot3")

(14)  get.ci.other(x=example.data$value, R=500, mode="mean")
(15)  get.ci.other(x=example.data$value, R=500, mode="perc.95")
(16)  get.ci.other(x=example.data$value, R=500, mode="top.5")
(17)  get.ci.other(x=example.data$value, R=500, mode="boost")
```

Protocol S1 Figure 1. *R* functions. *(1)* Clears *R* working environment. *(2)* Sets the directory of *R* working environment. *(3)* Imports example data set as an object in *R*. *(4)* Imports function code for subpopulation analysis in *R*. *(5)-(8)* Four different modes of subpopulation analyses where x is assigned the data to be analysed (in this case *example.data*). Modes *Default* and *Manual* represent interactive methods requiring mouse-clicking on a graph. Modes *Boxplot1.5* and *Boxplot3* run automatically once initiated. Mode *Default* requires two mouse-clicks on the approximate minimum and maximum of the large subpopulation, respectively. Mode *Manual* only requires one mouse-click on the approximate point of separation of the to subpopulations. *(9)* Mode *other* uses other methods of characterization namely mean, 95th percentile, boosted mean, and mean top five. *(6)-(13)* A function for each mode to determine confidence intervals reflecting consistency (reproducibility) of the method. Automatic modes *Boxplot1.5* and *Boxplot3* employ bootstrapping with R replicates. The Default setting of R is set to 500 but can be changed. Similarly, interactive modes *Default* and *Manual* employ resampled trials of x repetitions, with the Default set to 20 trials. *(14)-(17)* Confidence interval determination for mean, 95th percentile, boosted mean, and mean top five are based on bootstrapping with R = 500 rounds.

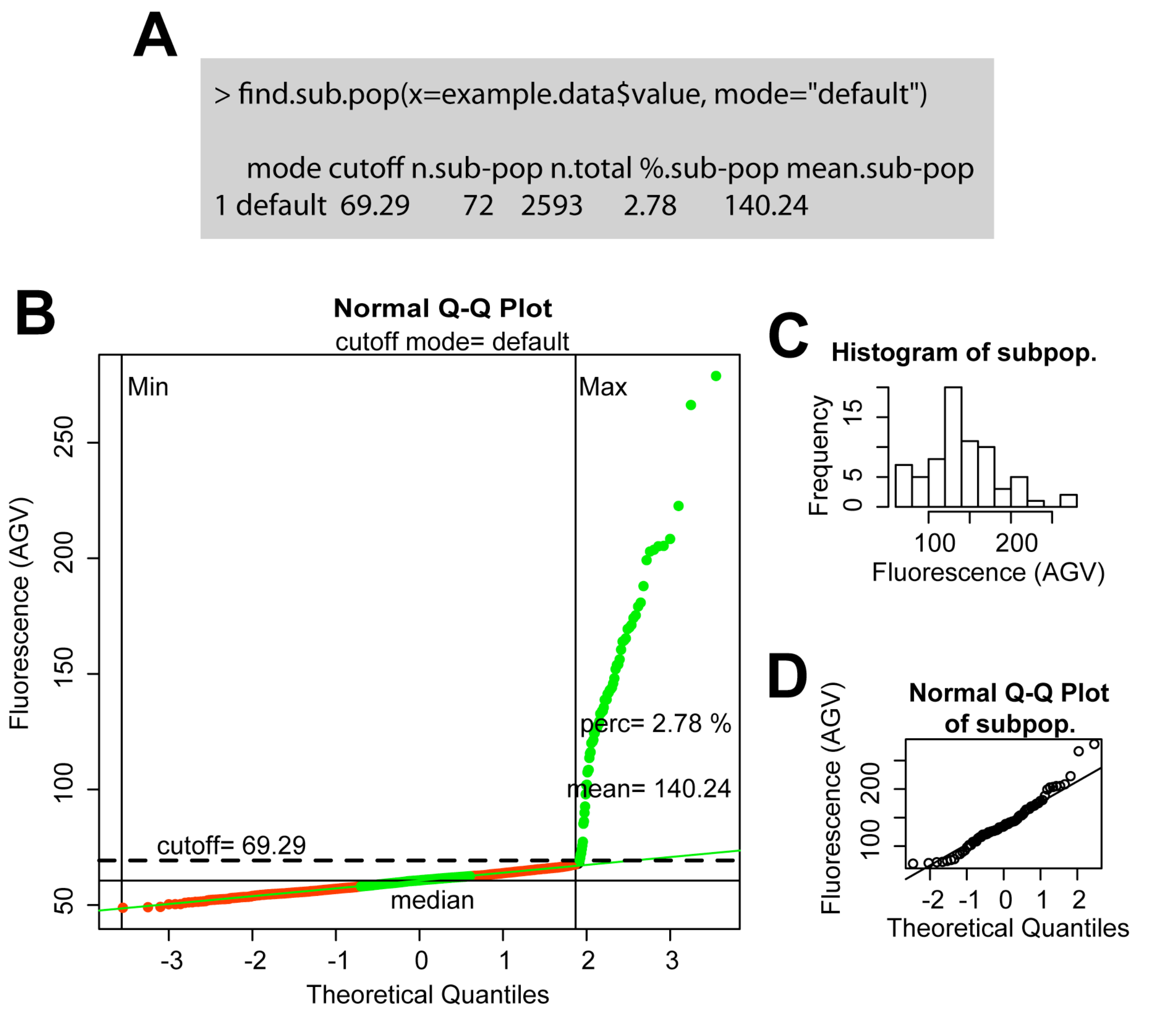

Protocol S1 Figure 2. Example of *R* output of the function *findsub(...)* with mode set to *Default* and *example.data* used as data set. (A) Command line output stating the cutoff value at which the small subpopulation was separated from the large subpopulation (cutoff), the size n of the small subpopulation (n.sub-pop) and the total population (n.total), the percentage of the small subpopulation (perc.sub-pop), and the mean value of the small subpopulation (mean.sub-pop). (B) Graphical output highlighting the manually placed minimum (Min) and maximum (Max) thresholds that encompass the estimated large subpopulation (vertical lines), its median (lower horizontal line), the points belonging to the IQR within the large subpopulation (green, vertical), the regression line fitted to the IQR points (green line), the cutoff value separating large and small subpopulation (dotted horizontal line), and the points belonging to the small subpopulation (green, vertical). (C) Histogram graphical output of the estimated small subsubpopulation. (D) QQ plot of the estimated small subpopulation.

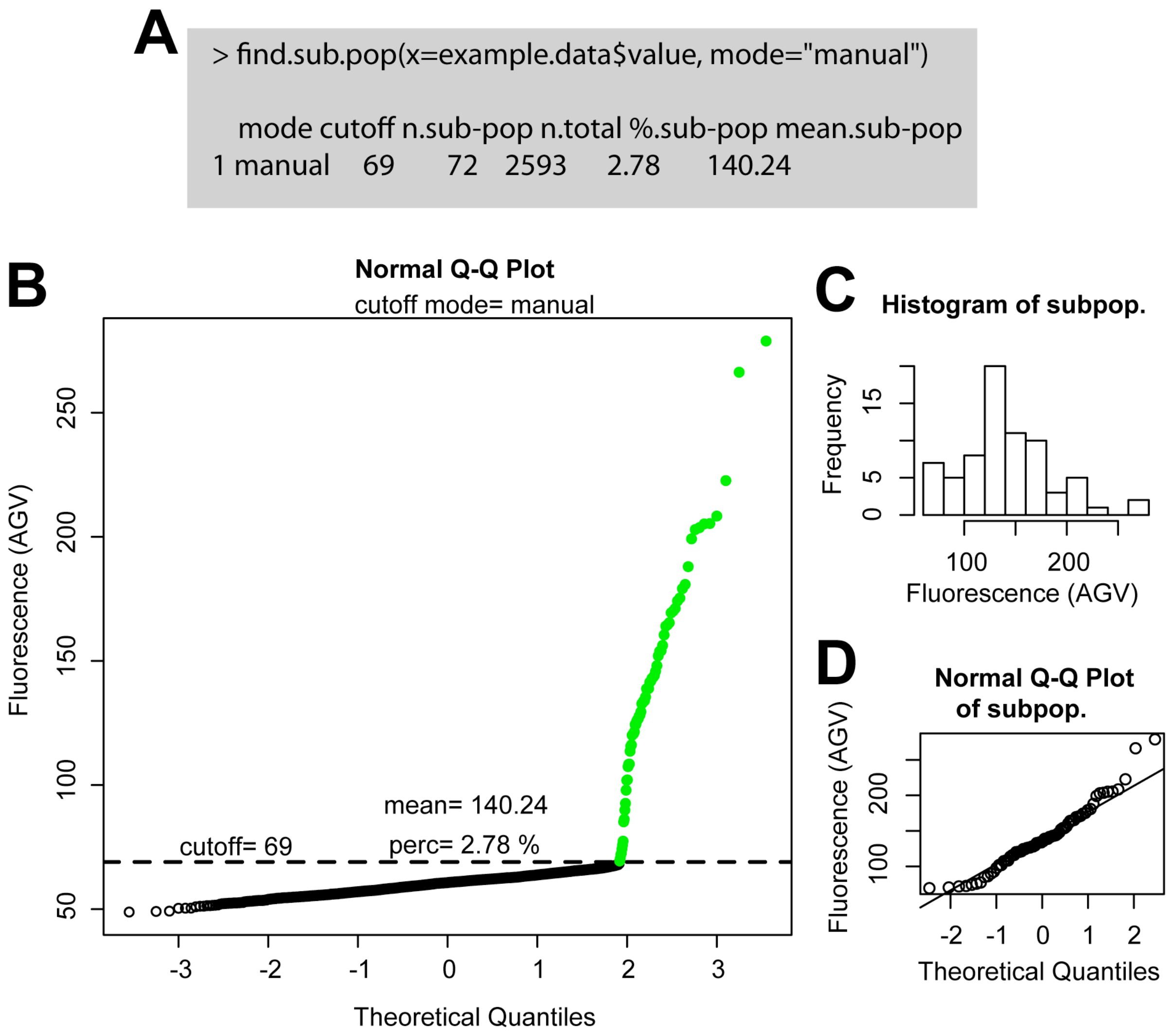

Protocol S1 Figure 3. Example of *R* output of the function *findsub(...)* with mode set to *Manual* and *example.data* used as data set. (A) Command line output stating the cutoff value at which the small subpopulation was separated from the large subpopulation (cutoff), the size of the small subpopulation (n.sub-pop) and the total population (n.total), the percentage of the small subpopulation (perc.sub-pop), and the mean value of the small subpopulation (mean.sub-pop). (B) Graphical output highlighting the manually placed cutoff separating large and small subpopulation (dotted horizontal line), and the points categorized as small subpopulation (green). (C) Histogram of the small estimated subpopulation. (D) QQ plot of the small estimated subpopulation.

A

```
> find.sub.pop(x=example.data$value, mode="boxplot1.5")

     mode cutoff n.sub-pop n.total %.sub-pop mean.sub-pop
1 boxplot1.5  69.39        72    2593      2.78       140.24
```

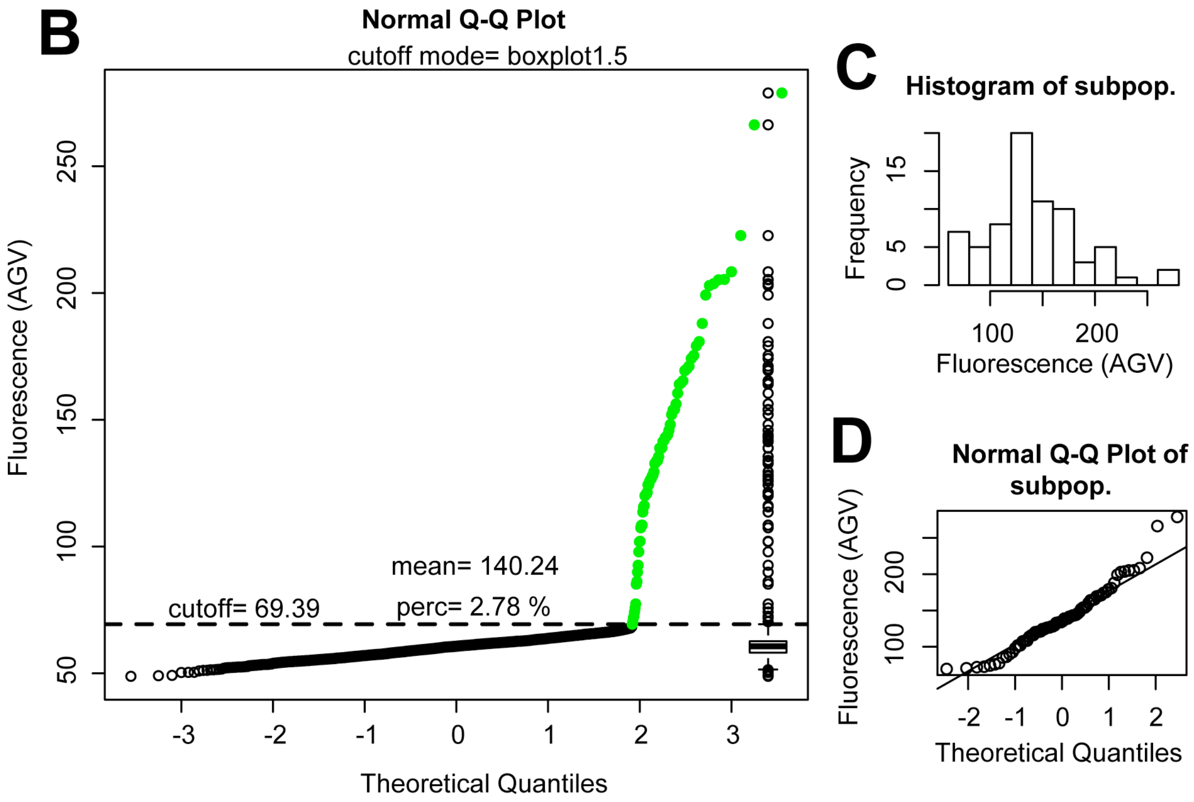

Protocol S1 3 Figure 4. Same as Figure 2 but with mode set to *Boxplot1.5*. Note that the cutoff value in this case is determined by how far the upper whisker of the boxplot extends, which is no more than 1.5 times the interquartile range from the box (upper quartile).

A

```
> find.sub.pop(x=example.data$value, mode="boxplot3")

     mode cutoff n.sub-pop n.total %.sub-pop mean.sub-pop
1 boxplot3  75.77       66    2593      2.55       146.41
```

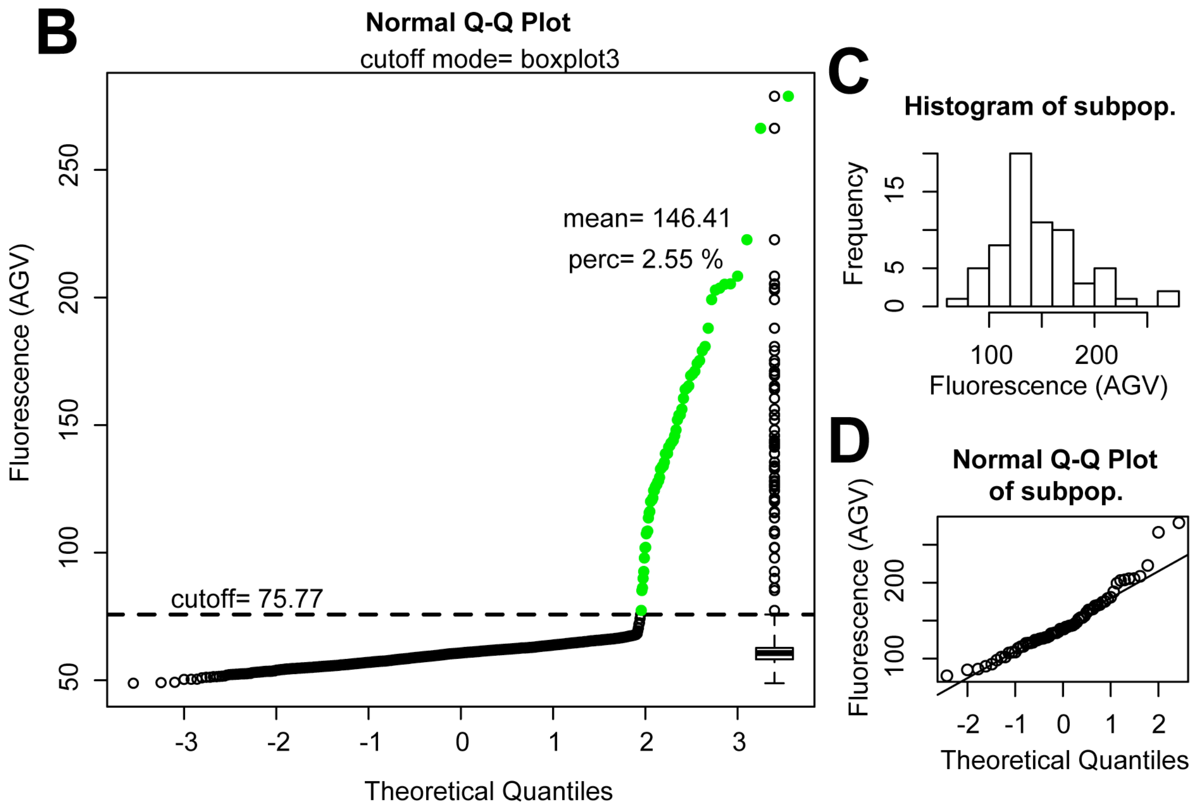

Protocol S1 Figure 5. Same as Figure 2 but with mode set to *Boxplot3*. Note that the cutoff value in this case is determined by how far the upper whisker of the boxplot extends, which is no more than 3 times the interquartile range from the box (upper quartile).

```
> find.sub.pop(x=example.data$value, mode="other")

  mean perc.95  perc.99   boost    top5
  62.5  66.2  146.0  64.1  107.5
```

Protocol S1 Figure 6. Same Figure 1 but with mode set to *Other*. Mode *Other* uses other methods of characterization namely mean (mean), 95th percentile (perc.95), 99th percentile (perc.99), boosted mean (boost), and mean top five (top5) of the total population, respectively.

A

```
> get.ci(x=example.data$value, trials=20, mode="default")
$quantiles
  2.5%   97.5%
2.25900 3.28275
$sd
[1] 0.2983887
$mean
[1] 2.796
$ci.95
[1] 2.211169 3.380831
$trials
 [1] 2.93 2.66 2.97 2.82 2.82 3.09 2.66 2.31 3.12 3.05
2.85 2.28 2.89 3.43 2.74 2.89 2.24 2.85 2.47 2.85
```

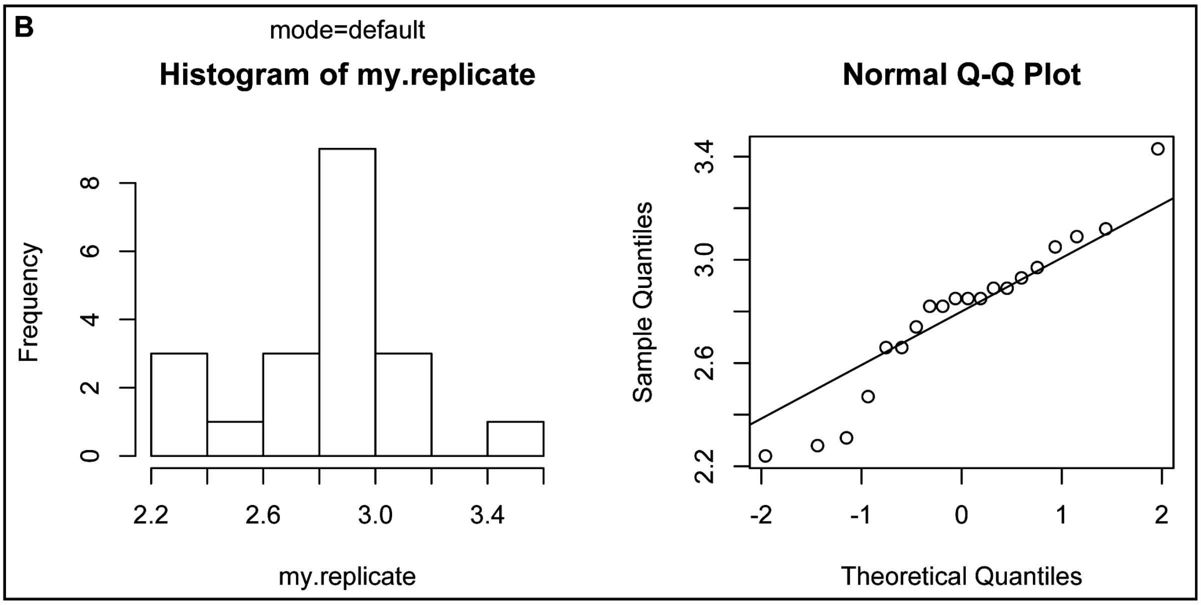

Protocol S1 Figure 7. Example of *R* output of the function *get.ci(...)* with mode set to *Default* and *example.data.txt* used as data set. (A) Command line output stating the 2.5 and 97.5 percentiles of the trial results ($quantiles), the standard deviation ($sd), the mean ($mean), and the 95% confidence interval ($ci.95). The latter was calculated according to $CI_{upper/lower} = mean \pm SD \times 1.96$, where $CI_{upper/lower}$ is the upper or lower confidence interval, *mean* is the population mean, and *SD* is the standard deviation of the boostrap results. Also shown are the individual results of applying *get.ci(...)* with mode set to *Default* on the re-sampled dataset ($trials). (B) Histogram graphical output (left) and QQ-plot graphical output (right) of the bootstrap results.

A

```
> get.ci(x=example.data$value, R=500, mode="boxplot1.5")
                    results
R                500.000000
Result             2.780000
Normal.low         2.148359
Normal.high        3.385401
Basic.low          2.090000
Basic.high         3.400000
Percent.low        2.160000
Percent.high       3.470000
```

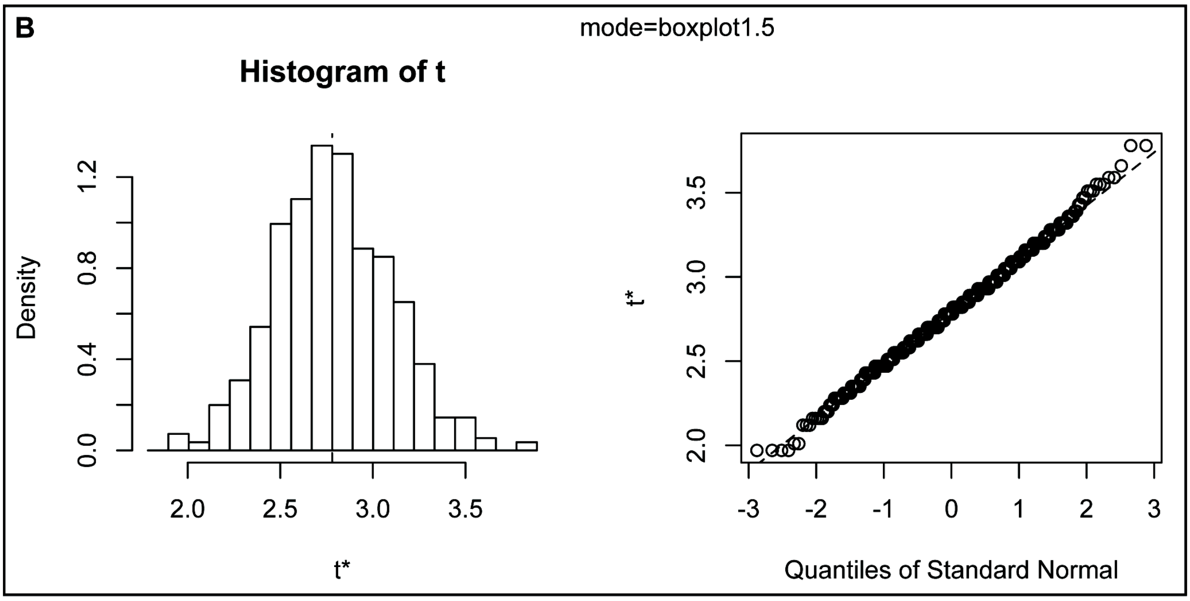

Protocol S1 Figure 8. Example of *R* output of the function *get.ci(...)* with mode set to *Boxplot1.5* and *example.data.txt* used as data set. (A) Command line output stating the number of bootstraps (R), the result of applying *get.ci(...)* with mode *Boxplot1.5* on the original dataset (Result), and confidence intervals according to different algorithms including the basic bootstrap method (Basic), the studentized bootstrap method (Normal) and the bootstrap percentile method (Percent). (B) Histogram graphical output (left) and QQ-plot graphical output (right) of the bootstrap results.

References

1. R Development Core Team: **R: A Language and Environment for Statistical Computing**. *R Foundation for Statistical Computing, Vienna, Austria ISBN 3-900051-07-0, URL http://wwwR-projectorg* 2009.
2. Canty A, Ripley B: **boot: Bootstrap R (S-Plus) functions**. *R package version 12-41* 2009.
3. Davison AC, Hinkley DV: **Bootstrap methods and their applications**. Cambridge: Cambridge University Press; 1997.
